# Decoding the evolutionary response to prostate cancer therapy by plasma genome sequencing

**DOI:** 10.1186/s13059-020-02045-9

**Published:** 2020-07-06

**Authors:** Naveen Ramesh, Emi Sei, Pei Ching Tsai, Shanshan Bai, Yuehui Zhao, Patricia Troncoso, Paul G. Corn, Christopher Logothetis, Amado J. Zurita, Nicholas E. Navin

**Affiliations:** 1 Department of Genetics, The University of Texas MD Anderson Cancer Center, Houston, TX USA; 2 MD Anderson Cancer Center UTHealth Graduate School of Biomedical Sciences, Houston, TX USA; 3 Department of Genitourinary Medical Oncology, The University of Texas MD Anderson Cancer Center, Houston, TX USA; 4 Department of Pathology, The University of Texas MD Anderson Cancer Center, Houston, TX USA; 5 David H. Koch Center for Applied Research of Genitourinary Cancers, The University of Texas MD Anderson Cancer Center, Houston, TX USA; 6 Department of Bioinformatics and Computational Biology, The University of Texas MD Anderson Cancer Center, Houston, TX USA

**Keywords:** Tumor evolution, Liquid biopsies, Non-invasive

## Abstract

**Background:**

Investigating genome evolution in response to therapy is difficult in human tissue samples. To address this challenge, we develop an unbiased whole-genome plasma DNA sequencing approach that concurrently measures genomic copy number and exome mutations from archival cryostored plasma samples. This approach is applied to study longitudinal blood plasma samples from prostate cancer patients, where longitudinal tissue biopsies from the bone and other metastatic sites have been challenging to collect.

**Results:**

A molecular characterization of archival plasma DNA from 233 patients and genomic profiling of 101 patients identifies clinical correlations of aneuploid plasma DNA profiles with poor survival, increased plasma DNA concentrations, and lower plasma DNA size distributions. Deep-exome sequencing and genomic copy number profiling are performed on 23 patients, including 9 patients with matched metastatic tissues and 12 patients with serial plasma samples. These data show a high concordance in genomic alterations between the plasma DNA and metastatic tissue samples, suggesting the plasma DNA is highly representative of the tissue alterations. Longitudinal sequencing of 12 patients with 2–5 serial plasma samples reveals clonal dynamics and genome evolution in response to hormonal and chemotherapy. By performing an integrated evolutionary analysis, minor subclones are identified in 9 patients that expanded in response to therapy and harbored mutations associated with resistance.

**Conclusions:**

This study provides an unbiased evolutionary approach to non-invasively delineate clonal dynamics and identify clones with mutations associated with resistance in prostate cancer.

## Background

Clonal evolution has been challenging to study in tumor tissue obtained from cancer patients, particularly in specimens collected from a single point in time [1–3]. While a small number of studies were able to collect serial core biopsies for genomic profiling [4], those studies still suffered from small amounts of tumor tissue availability and spatial sampling bias. Moreover, tumor biopsies are invasive clinical procedures with potential for complications and have significant costs [5, 6]. An alternative non-invasive approach involves using liquid biopsies, including circulating tumor cells [7–9] and cell-free DNA (cfDNA) [10–13]. Blood samples collected over time during the course of treatment provide a unique opportunity to infer tumor evolution [14]. cfDNA is particularly useful for clinical applications due to the logistical advantage of straightforward processing and the ability to cryostore materials for future analysis [15–17]. However, a major limitation has been that most cfDNA assays were developed to analyze targeted cancer gene panels and have therefore measured a limited number of CNAs and mutations [18–21]. Resolving intratumor heterogeneity and inferring clonal evolution requires measuring a large number of unbiased genomic markers, which targeted panels cannot provide.

A few studies have made initial progress towards performing unbiased exome sequencing of cfDNA in gastrointestinal cancers [22] and prostate cancer (PC) [10], or whole-genome copy number profiling of triple-negative breast cancer patients [23]. Building on those early studies, we have developed a method called PEGASUS (Plasma Exome and Genome Analysis by Size-selection Unbiased sequencing) to profile both genome-wide CNA and exome-wide mutations (~ 25,000 genes) simultaneously from archival cryostored plasma or serum DNA samples. In contrast to targeted methods [24–26], PEGASUS involves selection of small DNA fragments that contain higher tumor content relative to high molecular weight DNA from WBCs present in cryostored blood fractions. These characteristics make PEGASUS ideally suited for unbiased discovery of genomic markers and for the investigation of clonal evolution in response to therapy, when applied to longitudinal blood collections.

Comprehensive genomic studies have identified multiple recurrently altered genes (*AR*, *TP53*, *RB*, *SPOP*, *PTEN*, and others) and signaling pathways in metastatic prostate cancer (mPC) [27], but have also revealed the existence of many low-frequency variants and significant inter-patient heterogeneity. Moreover, PC is unique among solid tumors due to its dependence on androgen receptor-regulated pathways for progression and its high propensity to metastasize to the bone (often the only site of progression). The vast majority of previously untreated or castration-sensitive prostate cancer (CSPC) respond to initial androgen deprivation therapy (ADT), but the disease invariably adapts and progresses to lethal castration-resistance prostate cancer (CRPC) [28]. Treatment strategies for CRPC patients are limited and include advanced hormonal therapies and taxane-based chemotherapy, but no molecular markers have yet been established to guide their application and timing of treatment. Because of the bone dominance of metastatic prostate cancer (mPC), bone biopsies are most frequently needed for pathological and molecular characterization, but these are uncomfortable and difficult to perform procedures that often result in minute amounts of tumor tissue that is not suitable for genomic analysis. As a result, mPC presents a unique opportunity for liquid biopsy genomics to investigate heterogeneity and clonal evolution over time in the context of therapy.

## Results

### PEGASUS method

**Fig. 1:**
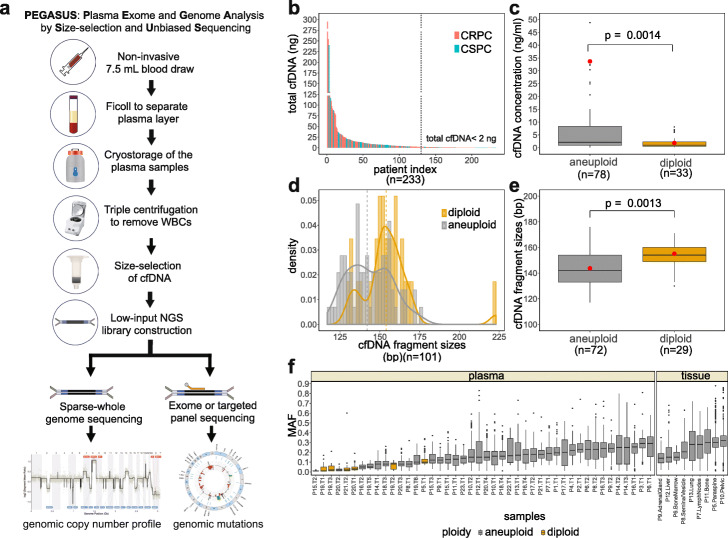
PEGASUS whole-genome plasma sequencing approach and molecular properties of cfDNA. **a** Workflow for the PEGASUS whole-genome plasma DNA sequencing approach. **b** Distribution of total cfDNA concentrations (nanograms) in the prostate cancer patients, with the dotted line showing the QC threshold (< 2 ng). **c** Comparison of cfDNA concentrations (ng/mL) between diploid and aneuploid genomic cfDNA profiles. **d** Distribution of cfDNA fragment sizes (bp). **e** Comparison of the distribution of cfDNA fragment sizes in basepairs between diploid and aneuploid genomic cfDNA profiles. **f** Distribution of the somatic mutation allele frequencies (MAFs) among the 23 plasma and 9 metastatic tissue samples. Significance in **c** and **e** was calculated using the Wilcoxon rank sum test. Red dots in **c** and **e** represent mean values

While prospective studies typically use Streck tubes to collect plasma, most archival plasma samples have historically been collected in EDTA tubes or subjected to a gradient treatment such as Ficoll prior to cryostorage. In contrast to Streck tubes (which include a fixative), the usage of EDTA/Ficoll medium often leads to contamination by high molecular weight DNA fragments that are released from the WBC during sample processing. To address this problem, PEGASUS was designed to isolate low molecular weight DNA fragments (< 1000 bp), which is done prior to the construction of low-input libraries for whole-genome sequencing (WGS) of copy number alterations (CNAs) and exome mutation profiling (Fig. 1a, the “Methods” section). After three rounds of high-speed centrifugation to remove residual WBC, the archival plasma samples are subjected to size selection by column purification. The remaining low molecular weight DNA is used for quality control (QC) to determine the plasma DNA concentration and molecular size of the DNA fragments. Samples that pass QC are used to construct low-input NGS libraries, which are split into two parallel reactions: (1) copy number profiling by WGS at sparse (0.1X) coverage depth and (2) exome sequencing at high coverage depth (150X tumor, 60X normal) to detect somatic mutations and indels (the “Methods” section). NGS libraries are generated in parallel from WBCs for exome capture to serve as a matched normal reference of germline variants to distinguish somatic mutations. This protocol can also be performed on fresh blood samples collected in Streck tubes.

### Molecular properties of cfDNA and clinical correlations

We performed QC of cfDNA in 233 cryostored archival plasma samples collected from PC patients at MD Anderson by measuring the size and concentration, including 79 patients with CSPC and 154 patients with CRPC (Fig. 1b). QC indicated that 130 of the 233 patients (55.8%) had sufficient cfDNA (≥ 2 ng total) for construction of NGS libraries using PEGASUS. The QC data showed a wide range in the total amounts of low molecular weight cfDNA (0–5280 ng) across the patients, including a subset of 29 patients (12.4% of the total) with very high amounts of total cfDNA (≥ 20 ng) (left side, Fig. 1b). The median cfDNA concentrations in CSPC (1.25 ng/mL) and CRPC patients (0.84 ng/mL) were not significantly different (*p* = 0.29, Wilcoxon test). However, 24 CSPC patients (30.4%) had < 0.5 ng of detectable plasma DNA compared to only 5 (3.2%) of the CRPC patients (Fig. 1b and Additional file 1: Fig. S2a), suggesting that a larger proportion of CRPC patients could potentially be used for genomic profiling using PEGASUS. We performed low-pass WGS of 101 plasma samples to understand how diploid and aneuploid genomic copy number profiles correlate with cfDNA properties. This data showed that cfDNA concentrations were significantly higher in patients with aneuploid genomes compared to patients with diploid plasma copy number (*p* = 0.00014, Wilcoxon rank sum test) (Fig. 1c).

Analysis of the cfDNA fragment sizes showed a mean molecular size of 146.6 ± 1.29 bp (SEM) across the patients, with a bimodal cfDNA size distribution (Fig. 1d, Additional file 1: Fig. S1). PC patients with diploid copy number had significantly (*p* = 0.0013, Wilcoxon rank sum test) larger cfDNA fragment sizes (mean 154 ± 1.66 bp) compared to patients with aneuploid profiles (mean 142 ± 2.91 bp) (Fig. 1d, e). These numbers are consistent with previous reports on plasma DNA fragment size distributions for circulating tumor DNA (ctDNA) compared to cfDNA that was isolated from normal cells [29, 30], suggesting that the tumor cells are not shedding large amounts of DNA into the plasma. Notably, a subset of patients had additional peaks at double and triple the mean cfDNA fragment size, possibly indicating that the DNA was protected by multiple nucleosomes, rather than a single nucleosome detected in most PC patients (Additional file 1: Fig. S1a-d).

While most of the 233 patient samples used for QC were from single-timepoint blood samples, there were 9 patients with matched metastatic tissues and 12 patients with longitudinal blood samples collected during therapy that were selected for genomic CNA profiling and exome sequencing by PEGASUS. To test the variability in ctDNA concentration after isolation, we performed 4 experimental replicates from 4 test plasma samples, which showed only minor variations in the final concentrations of ctDNA (Additional file 1: Fig. S1e). Analysis of the somatic exome mutation allele frequency (MAF) showed a median MAF of 0.17 for the aneuploid cfDNA samples, which was slightly lower than the MAF detected in matched metastatic tumor tissues (median 0.3) (Fig. 1f). Notably, plasma DNA with diploid genomic copy number profiles showed the lowest MAF (median 0.08), suggesting that the contribution of cfDNA from normal cells was high in these patients, with limited ctDNA.

### Clinical correlations with plasma DNA properties

**Fig. 2:**
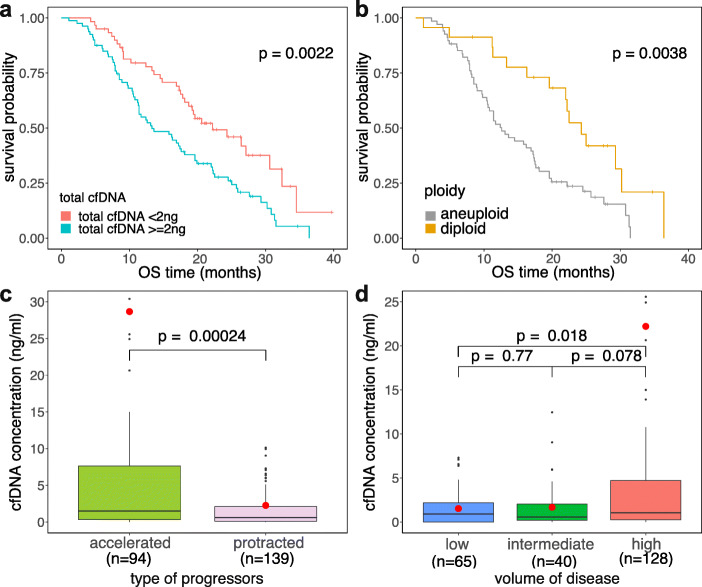
Survival analysis and correlation of cfDNA concentration with clinical features. **a** Kaplan-Meier overall survival plot for prostate cancer patients with total plasma DNA < 2 ng and total plasma DNA ≥ 2 ng. **b** Kaplan-Meier overall survival plot for patients with aneuploid and diploid cfDNA CNA profiles. **c** Comparison of the distribution of cfDNA concentration between accelerated and protracted progressors. **d** Distribution of cfDNA concentrations (ng/mL) between patients with low, intermediate, and high volume of disease. Significance for the survival analysis in **a** and **b** was calculated with the log-rank test, while the significance of the box plots in **c** and **d** was calculated using the Wilcoxon rank sum test. Red dots in **c** and **d** represent mean values

Survival analysis revealed that CRPC patients with total cfDNA < 2 ng had significantly longer overall survival (OS) than patients with total cfDNA ≥ 2 ng (median 22.2 vs 13.3 months, *p* = 0.0022, log-rank test) (Fig. 2a, Additional file 1: Fig. S2). Furthermore, patients with diploid cfDNA CNA profiles had longer OS than patients with aneuploid cfDNA CNA profiles (median 21.05 vs 12.6 months, *p* = 0.031, log-rank test) (Fig. 2b, Additional file 1: Fig. S2). We next investigated associations between cfDNA concentrations and clinical parameters. Patients with accelerated PC growth had higher cfDNA levels (*n* = 94) compared to those with protracted kinetics of progression (*n* = 139; *p* = 0.00024, Wilcoxon rank sum test) (Fig. 2c). Furthermore, PC patients with high disease volume (*n* = 128) had significantly higher cfDNA levels compared to patients with low disease volume (*n* = 65; *p* = 0.018, Wilcoxon rank sum test) (Fig. 2d). Notably, cfDNA concentrations were not significantly different (*p* = 0.4642, Wilcoxon rank sum test) in patients with histologic low grade groups 1–2 (*n* = 54) compared to high grade groups 3–5 (*n* = 164) on diagnosis (Additional file 1: Fig. S2d). By comparing local and distant metastases, PC patients with bone (*n* = 132) or visceral metastasis (*n* = 41) had respectively higher cfDNA concentrations than patients with lymph node metastasis only (*n* = 30; *p* = 0.042 and *p* = 0.0099 for bone and visceral metastasis, respectively, vs lymph node) (Additional file 1: Fig. S2e). Similarly, PSA levels had a low correlation with cfDNA concentrations (*R* = 0.12, *p* = 0.073) (Additional file 1: Fig. S2f). Finally, we utilized all the clinical and genomic factors as predictors for the OS and progression-free survival (PFS) using the univariate and multivariate Cox regression model in a subset of 70 patients that had consistent clinical variables available for all of the parameters we tested. Based on the survival analysis, we found that ploidy has significant independent and joint predictive power for OS, with significant joint predictive power for PFS (Additional file 1: Table S1). Collectively, these data suggest that higher cfDNA in the plasma and genomic aneuploidy associate with faster mPC progression and a shorter survival of PC patients.

### Whole-genome profiling of single-timepoint samples

**Fig. 3:**
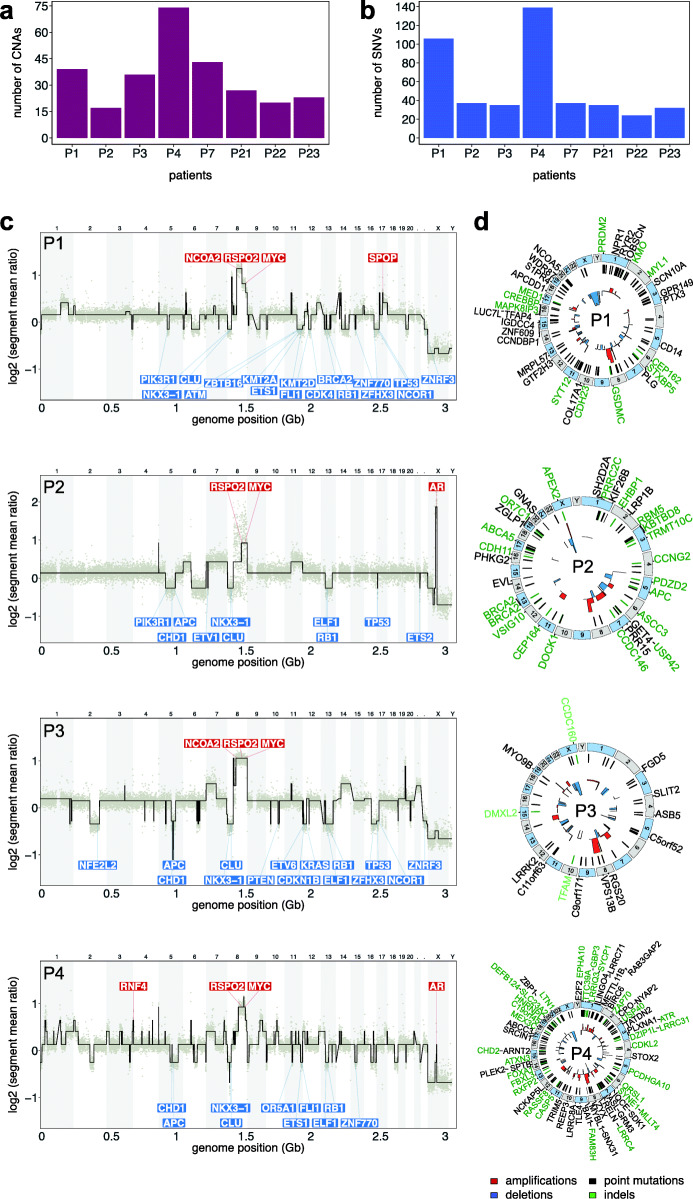
cfDNA sequencing of single-timepoint samples. **a** Global number of CNAs detected in each of 8 patients. **b** Mutation burden quantified from exome data of 8 patients, including all exonic mutations. **c** Genomic copy number ratio and segmentation plots of plasma DNA from 4 prostate cancer patients, with annotations of prostate cancer genes amplified shown in red boxes and lost shown in blue boxes. **d** Circos plots of CNAs, indels, and point mutations for the plasma DNA of the 4 patients, with prostate cancer genes annotated in the outer ring

We applied PEGASUS to obtain integrated genomic CNA and exome mutation profiling of single-timepoint plasma samples from 8 CRPC patients (Fig. 3, Additional file 1: Fig. S3). These patients had a mean 34.8 ± 6.49 (SEM) CNAs and 55.6 ± 14.99 (SEM) point mutations and 16 ± 3.15 (SEM) insertion-deletions (indels). However, two of the patients (P1, P4) had a much higher mutation burden (106 and 139 mutations, respectively) (Fig. 3a). Recurrent CNAs identified included amplifications in *MYC* (8q) in all 8 patients and in *AR* (Xq) in 4 patients, as well as losses in *RB1* (13q) in 7 patients, *TP53* (17p) in 4 patients, and *APC* (5q) in 3 patients (Fig. 3c, Additional file 1: Fig. S3a). Point mutations were identified in *GNAS*, *NCOA5*, *EVL*, and *BIRC6*, while indels were found in *BRCA2*, *APC*, *TP53*, and *FOXA1* among other cancer genes. Our unbiased analysis also identified CNAs and mutations in genes that have not previously been associated with mPC progression (Fig. 3c, Additional file 1: Fig. S3b). The overall distribution of the CNAs among the 8 single-timepoint samples shows a negative binomial distribution, with a majority of the CNA genomic sizes < 50,000 kb (Additional file 1: Fig. S3c). On a per-patient basis, the chromosome length of the CNAs are generally < 50,000 kb while a subset of CNAs have larger size distributions (Additional file 1: Fig. S3d). This data demonstrates the technical feasibility of using PEGASUS to perform unbiased genomic profiling and detect both recurrent and infrequent PC aberrations in cfDNA.

### Tumor DNA concordance in plasma and metastatic tissue

**Fig. 4:**
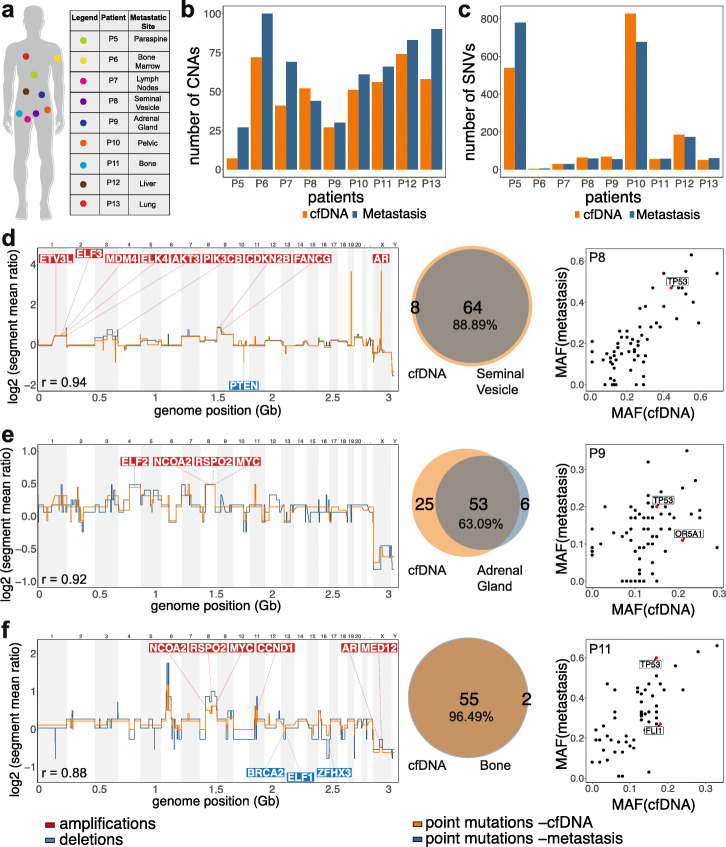
Concordance of plasma DNA and metastatic tissue samples. **a** Metastatic organ site location of the matched tissue samples. **b** Total number of CNAs identified across the 9 metastatic patients. **c** Mutation burden quantified from exome data of 9 patients, including both non-synonymous and synonymous exonic mutations. **d**–**f** Genomic copy number data and exome mutations for 3 patients with matched metastatic tissue samples, with prostate cancer genes labeled

To investigate the concordance of genomic events between the cfDNA and metastatic tumor tissues, we applied PEGASUS to 9 CRPC patients with matched tissue specimens obtained from different metastatic organ sites (Fig. 4a). Global genomic analysis showed that the CNA burden in plasma and metastatic tumor tissue was highly correlated (mean *r* = 0.9) in most patients (P8–P12) (Fig. 4b). However, in 4 patients (P5, P6, P7, P13), the CNA burden in the plasma was lower than the corresponding tumor tissue (mean *r* = 0.6), suggesting that some tumor clones in the metastatic sites did not shed sufficient DNA into the blood for detection (Fig. 4b). Similarly, the total mutation burden was highly concordant between the matched cfDNA and metastatic tissues (51–96%), including two patients (P5 and P10) who had a very large number of somatic mutations (> 200) in both the blood and tissue, consistent with a hypermutator genotype [31, 32] (Fig. 4c). In P10, deletions in both *MSH2* and *MSH6* were detected, while in P5, an *MLH1* deletion was identified, which may have contributed to the high mutation burden in these patients (Additional file 1: Fig. S4a; Additional file 1: Fig. S4d).

Further analysis showed that most CNAs, including amplification in *AR* and deletion in *PTEN* in P8, amplifications in *ELF2* and *MYC* in P9, and amplifications in *MYC* and *CCND1* in P11, were concordant in the plasma and tumor tissues (Fig. 4d–f). However, we also found a small number of discordant CNAs that were exclusive to the metastatic tumor tissue (P6) or the cfDNA (P8, P9, P12, and P13). P6 had a focal amplification in 12p21 (3.72 mb, including *KRAS*) in a bone metastasis site that was not detected in the cfDNA. In P8, a focal amplification in 19p (1.52 mb) was not detected in the matched seminal vesicle metastasis, while in P9, a focal amplification in 11p (6.7 mb) was not detected in the corresponding adrenal gland metastasis. Similarly, P12 had amplifications in 8q (*MYC*) and Xp (*AR*), while P13 had two focal amplifications in Xp (*AR* and *ELK1*), which were not detected in the matched metastatic tissues (Additional file 1: Fig. S4f).

Analysis of the MAFs showed a linear correlation for most somatic mutations, but also identified mutations that were exclusive to either the plasma DNA or the metastatic sites (Fig. 4d–f, Additional file 1: Fig. S4a-S4f). However, most driver mutations including *TP53*, *AR*, *ATM*, *SPOP*, *FLI1*, and *OR5A1* were detected in both the plasma DNA and tissues. Overall, the matched tissue data showed a high concordance in CNAs and point mutations between cfDNA and metastatic tissues, suggesting that the ctDNA is highly representative of many of the genomic aberrations detected in tumor tissues.

### Genomic response to therapy in plasma DNA

**Fig. 5:**
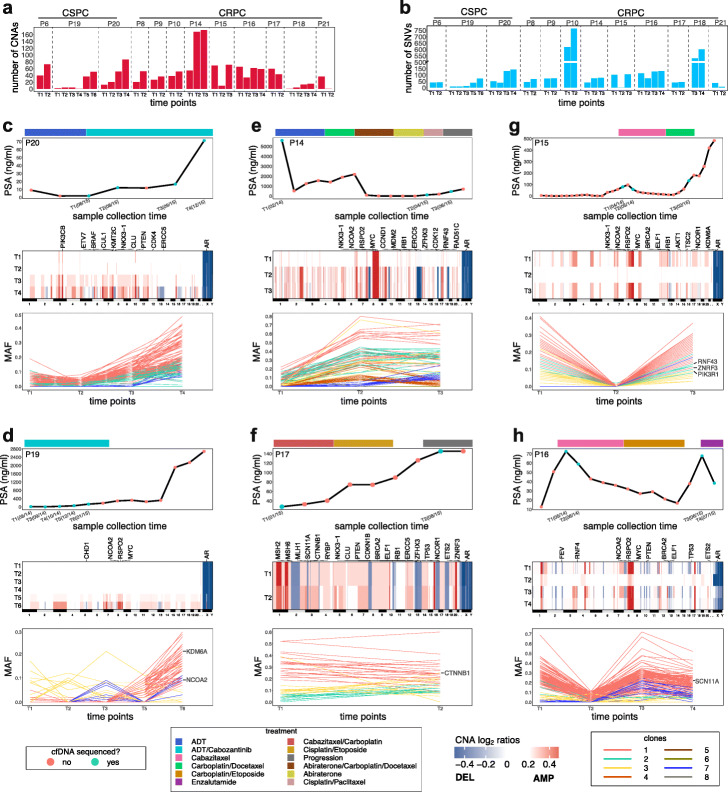
Genomic response in longitudinal cfDNA samples. **a** Total number of CNAs detected in longitudinal timepoints from 12 patients. **b** Mutation burden quantified from exome data of 12 patients, using all exonic mutations. **c**–**h** Plots of treatment schedules and therapeutic agents against changes in PSA levels (ng/mL) in 6 patients, with genomic copy number heatmaps and exome MAF plotted below for each timepoint. **c**, **d** CSPC patients with increasing mutations and CNAs. **e**, **f** CRPC patients with minor changes in mutations in CNAs during treatment. **g**, **h** CRPC patients with transient genomic response. Colors in mutation line plots represent different clones inferred by CITUP (the “Methods” section), while blue colors in PSA plots represent timepoints that were sampled for sequencing analysis

We next applied PEGASUS to plasma DNA samples collected serially (2–6 timepoints) from 12 patients, including 9 CRPC patients (P8, P9, P10, P14, P15, P16, P17, P21) and 3 CSPC patients (P6, P19, P20), to study genomic response to therapy (Additional file 1: Table S2). The 3 CSPC patients were treated with ADT and a tyrosine kinase inhibitor (cabozantinib), while the CRPC patients received different chemotherapeutic and androgen-targeted agents (Fig. 5, Additional file 1: Table S2). From each timepoint, genomic CNA profiling and exome sequencing (mean depth 125X) were performed on the cfDNA, as well as matched normal PBMC samples (mean depth 77X) to detect germline variants.

On average, 126 somatic mutations (Fig. 5a) and 21 CNAs (Fig. 5b) were detected per patient, consistent with previously reported values in advanced prostate tumors [20, 31, 33, 34]. Integrated analysis revealed that the CNA burden (Fig. 5a) and point mutation burden (Fig. 5b) did not change substantially between the pre-treatment and post-treatment timepoints for most CRPC patients. However, in the mid-treatment timepoints, the CNA burden and mutation burden decreased substantially in several patients (P15, P16, P21), suggesting a transient genomic response to therapy. Notably, two CRPC patients (P10, P18) had high levels of somatic mutations (mean 601.5 SNVs), consistent with a hypermutator genotype [31, 32].

To investigate genomic response to therapy, we compared the plasma CNA profiles and exome MAF with the PSA levels for each patient (Fig. 5c–h, Additional file 1: Fig. S5a-S5f). In 2 CSPC patients (P19 and P20), we found increasing numbers of CNAs and mutations during treatment that corresponded to increasing PSA levels. In P6, who was hormone-naïve at the time of his first blood collection, an aneuploid profile was detected at both timepoints and accumulated CNAs at T2 as the disease progressed to CRPC. A comparable increase in mutation burden over time was found in one CRPC patient (P18) receiving chemotherapy (Additional file 1: Fig. S5d). In these patients, the increase in genomic aberrations over time was likely due to increasing tumor purity due to increasing tumor volume and/or increasing tumor DNA shedding, rather than acquisition of new CNAs in response to treatment, since the total number of chromosome breakpoints did not change over timepoints.

In the 9 CRPC patients, complex aneuploid rearrangements and high MAF were detected at the baseline plasma sample (prior to treatment) and at the mid- or post-treatment timepoints. In P9, P10, P14, and P17, the complex aneuploid rearrangements and MAF were pre-existing before therapy and persisted through all of the timepoints analyzed, suggesting the tumors were intrinsically resistant to the therapies administered (Fig. 5e–f, Additional file 1: Fig. S5c-S5d). In contrast, P15, P16, and P21 showed transient genomic responses to therapy in the mid-treatment timepoints, in which the genomic CNA profiles approached a near-diploid state and the MAF decreased substantially (Fig. 5g, h, Additional file 1: Fig. S5e). However, these responses were temporary, and the complex CNA profiles and high MAF returned in the later timepoints which is consistent with the change in PSA values.

**Fig. 6:**
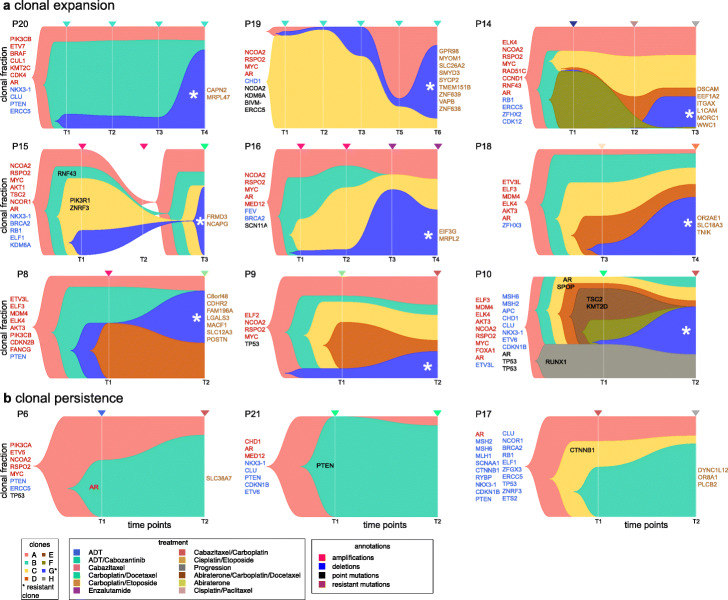
Clonal evolution in response to treatment inferred from cfDNA. Plots of clonal lineages and frequency changes over time and in response to treatment for 12 patients. CNAs and mutations are labeled in the inferred lineages, as well as significant mutations identified in the resistant clones (blue, asterisk) on the right-hand side. **a** Patients in which subclones were identified that expanded in response to therapy. **b** Patients in which clonal frequencies were persistent and remained stable during treatment

In most patients, all of the CNAs were detected at the pre-treatment timepoint and did not change during treatment, suggesting the CNA had been acquired at earlier stages of tumor progression, prior to treatment (Fig. 6). This included amplifications in *AR* (10/12 patients), *MYC* (7/12 patients), and *NCOA2* (7/12 patients) and deletions in *PTEN* (5/12 patients), *RB1* (3/12), and *BRCA2* (3/12) (Fig. 5, Additional file 1: Fig. S5). The only exception was in P6 who acquired an *AR* amplification in response to ADT (Additional file 1: Fig. S5a).

In contrast to the CNAs, many mutations underwent dynamic changes in MAFs in response to therapy and the rise or drop of MAFs was fairly consistent with the rise or drop in PSA levels. In the CSPC patients, the MAFs increased (P19, P20) or remained stable (P6) during treatment timepoints. In the CRPC patients, the MAFs persisted with only minor frequency changes in patients with intrinsically resistant disease (e.g., P14, P17) or showed transient decreases in patients that were responding to therapy (e.g., P15, P16). This included point mutations in known PC driver genes, including *TP53* (P6, P9, P10), *AR* (P10), *SCN11A* (P16), *NCOA2* (P19), and other genes (Fig. 5, Additional file 1: Fig. S5). We also identified subclonal mutations that emerged during treatment, including mutations in *PTEN* (P21), *RNF43*, *PIK3R1* and *ZNRF3* (P15), *SPOP*, *RUNX1* and *AR* (P10), and *CTNBB1* (P17). Many other somatic mutations were detected in genes not associated with PC, and increased or decreased in MAFs during treatment, suggesting a potential association with sensitivity or response to the therapeutic agents.

### Clonal evolution in response to therapy

To infer clonal evolution and identify subclones associated with resistance, we integrated the genomic CNA and exome mutation data from the serially collected plasma samples of the 12 patients. The confounding effects of tumor purity on CNA and MAF were normalized, and subclones were inferred across multiple timepoint samples using PyClone2 [35] and CITUP [36] (the “Methods” section, Additional file 1: Fig. S6). We identified multiple subclones (range 2–8) in the 12 patients and identified dynamic changes in clonal frequencies in response to therapy (Fig. 6). In 9 patients, minor subclones (1–21%) were identified that expanded in response to therapy (Fig. 6a), while in 3 patients (P6, P21, and P17), the most prevalent clone retained a similar frequency during treatment, suggesting that the dominant clone was intrinsically resistant to the treatment (Fig. 6b).

The clonal frequency data identified rare subclones that dynamically expanded during therapy and were therefore associated with resistance (Fig. 6a). For example, in P20, a minor subclone (clone G) with 7–11% clonal frequency in the initial timepoints (T1–T3) expanded to 75% at the final treatment timepoint (T4). This subclone harbored significant damaging mutations (SIFT< 0.05, POLYPHEN > 0.85) in two genes: *CAPN2* and *MRPL47*. In P18, a rare subclone (clone G) expanded from 6% at T3 to 53% at T4 and harbored three significant mutations: *SLC18A3*, *TNIK*, and *OR2AE1*. In P16, a rare subclone (clone G) expanded from 3 to 4% at the initial timepoints (T1–T2) to 41–57% at the final timepoints (T4–T5) and harbored significant mutations in *EIF3G* and *MRPL2*. In total, 9 patients were identified in which a minor subclone expanded during treatment, suggesting association with resistance.

The resistant subclones harbored a total of 36 mutations with significant SIFT (< 0.05) and POLYPHEN (> 0.85) functional impact scores (Additional file 1: Table S3). Notably, this data did not identify any recurrent genes associated with resistance; however, several recurrent gene families were identified, such as the solute carrier transporters (*SLC12A3*, *SLC38A7*, *SLC18A3*, and *SLC26A2*) and the mitochondrial ribosomal protein genes (*MRPL2* and *MRPL47*). Despite the lack of recurrence, many of the genes identified in the resistant clones have been previously implicated in PC progression or therapeutic resistance. For example, *NCAPG* detected in P15 is part of the condensin complex and has been related to CRPC pathogenesis [37, 38]. *LGALS3* detected in P8 is a member of the galectin proteins involved in apoptosis, immunity, and adhesion, and was linked to treatment resistance in PC [39, 40]. *CAPN2* detected in P20 is an intracellular cysteine protease and has been shown to promote cell proliferation and invasion in CRPC cell lines [41]. Furthermore, *WWC1*, which was identified in P14, was found to be upregulated in antiandrogen-resistant PC cell lines [42].

We further investigated if the resistance-associated mutations identified in this study were correlated with poor survival in the TCGA datasets (*N* = 3811 patients), and found significant associations with poor survival for *CAPN2*, *CDHR2*, *LGALS3*, *L1CAM*, *MORC1*, *EIF3G*, *OR8A1*, *TNIK*, and *SMYD3* (adjusted *p* value < 0.05, Benjamini-Hochberg correction) (Additional file 1: Table S3, the “Methods” section). Collectively, these data suggest that the evolutionary analysis of clonal dynamics in cfDNA may be useful for delineating intratumor heterogeneity and identifying resistant clones and mutations associated with therapy response and resistance in prostate cancer patients.

## Discussion

Here, we report the development of an unbiased whole-genome sequencing approach for cryostored plasma DNA and its application to study clonal diversity and evolution in response to therapy in PC patients. In contrast to targeted cfDNA methods [21, 43], PEGASUS was designed to perform unbiased genome-wide profiling of CNAs and mutations, which are necessary to infer evolutionary dynamics over time and identify clones associated with response to therapy.

In several patients, we compared plasma DNA directly to matched tissue samples from metastatic organ sites. Our findings suggest that the genomic aberrations identified in cfDNA are highly representative of the metastatic tissue sites, but also that the cfDNA also contains mutations that are not present in the matched metastatic tissues. These additional mutations in the plasma may originate from other metastatic foci or micrometastases that were not profiled in this study. Based on this data, we speculate that cfDNA provides a more holistic representation of a patient’s cancer genomic aberrations, across many of the primary and metastatic tumor sites, compared to core biopsy samples that reflect a limited spatial area in a single tissue site.

We further applied PEGASUS to analyze serial plasma samples collected from PC patients that received different combinations of hormonal and chemotherapy treatments. Our data showed that complex aneuploid rearrangements remained highly stable during treatment, with few or no new CNA acquired during this time. This data suggest that CNAs are likely to have occurred early in tumor evolution and may be related to intrinsic (rather than acquired) resistance of the tumor cells. In contrast, the MAF in the cfDNA underwent dynamic changes in response to treatments. In most patients, clones present in low frequency in the pre-treatment timepoints expanded during therapy and harbored mutations associated with resistance. Many of the genomic aberrations in the genes identified (*N* = 9) in the resistance clones were correlated with poor survival in larger cohorts of PC patients in TCGA. However, most mutations identified in the resistant clones were not recurrent across patients, a possible reflection of the heterogeneous nature of the treatments or alternatively of diverse mechanisms of resistance. An exception was several recurrent mutations in solute carrier transporter genes (SLC) and mitochondrial ribosomal protein (MRP) gene families. The SLC genes are of considerable interest from a therapeutic standpoint, since they are involved in the uptake and transport of drugs into cells and therapeutic resistance [44]. Future studies will be needed to functionally validate these mutations and understand their potential role in therapy resistance in PC patients.

Several clinical parameters identified correlated with increased plasma cfDNA concentrations, including the presence of aneuploid genomes, increased disease volume, accelerated progression, and poor OS. For cancers progressing in sites that are difficult to biopsy and/or that are unlikely to yield enough tumor cells to allow for genomic analysis (e.g., bone), PEGASUS may provide an invaluable profiling tool to discover genomic biomarkers associated with disease behavior and drug sensitivity. Global genomic features such mutation burden measured in the cfDNA may have clinical utility for identifying patients with increased neoantigens that are ideal for treatment with immune checkpoint inhibitors. Furthermore, the CNA burden or aneuploid aberrations detected in cfDNA may be used to detect tumors with homologous recombination deficiency (HRD) in patients who may benefit from agents targeting DNA damage repair defects such as PARP inhibitors [45, 46]. Indeed, our data suggest that the detection of aneuploid copy number profiles in plasma DNA is an indicator of poor survival in PC patients, as are higher concentrations of cfDNA in the blood.

The use of PEGASUS to study genomic aberrations in plasma DNA has a few limitations. Foremost, because the genomic profiling is unbiased and broad by the assay design, it requires significant sequencing coverage (e.g., 150X) and higher cost, compared to targeted panels, which also have a higher sensitivity for detection of rare mutations. Another limitation is that the approach is more suitable for patients with advanced and metastatic disease and is unlikely to have utility in the detection of early disease, where the concentration of ctDNA in the plasma is very low. This was an issue in the initial timepoints analyzed in the CSPC patients, where only diploid genomes were detected prior to progression to CRPC disease. In such cases, the use of targeted plasma DNA sequencing panels (e.g., Guardant360, Oncomine) would be more appropriate to increase detection sensitivity.

In closing, we expect that PEGASUS will have a myriad of applications in cancer research, particularly in the discovery and identification of genomic mechanisms of response and resistance to anticancer therapy in large patient cohorts, where longitudinal plasma samples have previously been collected and cryostored for achieving purposes and long-term clinical outcome data is available. Our approach will be particularly useful in the non-invasive genomic profiling of solid tumor tissues that are challenging to biopsy (e.g., kidney, brain, lung, bone). We expect that these longitudinal genomic analyses will reveal basic mechanisms of response and disease resistance and may lead to new clinical assays that can monitor response to therapeutic agents and guide treatment decisions in cancer patients.

## Conclusions

This study shows that unbiased whole-genome sequencing of plasma DNA from prostate cancer patients can detect mutations and copy number alterations that can be used to infer clonal dynamics and genome evolution longitudinally in response to treatment. By computationally integrating this data over multiple timepoints, we use an evolutionary approach to identify clones that harbor mutations associated with therapeutic resistance.

## Methods

### Patient clinical data and sample information

All patients in this study were treated for prostate cancer at the University of Texas MD Anderson Cancer Center (Houston, TX) and provided informed consent per an Institutional Review Board-approved prospective protocol. Two patient cohorts were included: (i) newly diagnosed metastatic and hormone-naïve (CSPC) (including patients participating in clinical trials NCT01409200 or NCT01630590) and (ii) CRPC (including patients participating in clinical trial NCT01505868). All blood plasma and tissue samples were collected before initiation of systemic treatment, while progressing on therapy by PSA or radiologic criteria, or while on systemic treatment as indicated. Patients were prospectively followed from the time of inclusion until the last visit or death. Matched metastatic tissue samples were obtained as FFPE blocks from the respective patients. Patients were classified as aneuploid or diploid based on the whole-genome copy number data from the plasma. Patients with worsening performance status, pain, or other symptoms related to tumor growth in the 6 weeks prior to the blood specimen collection, and/or with development of > 2 new metastatic lesions in a single site or new non-nodal organ site extension in the previous 3 months, were classified as “accelerated progressors”; all other patients were defined as “protracted progressors.” Patients were classified as “high disease volume” if they had > 10 focal bone metastases or equivalent and/or tumor mass > 4 cm at any site, and/or extension to at least three organ sites with one lesion at least 2 cm in diameter; “low disease volume” if patients had ≤ 4 bone metastases with or without extension to lymph nodes up to 2 cm in diameter; all others were categorized as “intermediate disease volume” patients. A sample was classified as aneuploid if the CNA profile contained a large number of segments (> 45) and had a deviation from the median segmentation value (> 0.03) that was not explained by technical noise. If the sample had an intermediate number of segments (between 25 and 45), it was considered aneuploid if it had a deviation from the median segmentation value (> 0.03) and a large segment size or high segmentation value. All other samples were classified as diploid.

### Isolation of plasma DNA from blood plasma and quality control

Blood (approximately 7.5 mL) from the prostate cancer patients was collected in Ficoll tubes (catalog no. 362753). After gentle inversion, tubes were centrifuged at 1800*g* for 15 min at room temperature. The plasma layer was separated from the nucleated PBMC cell layer and centrifuged three times at 1500*g* for 10 min to remove contaminating cells. The PBMC layer was used to isolate genomic DNA, which was sequenced separately to identify germline variants (see genomic DNA isolation and quality control section). In cases where fresh blood samples were not available, frozen plasma stocks stored at − 80 C were thawed and centrifuged at 16,000*g* three times to remove all cryoprecipitates. Low molecular weight plasma DNA was purified by size selection (< 1000 bp) from high molecular weight DNA using the QIAamp® Circulating Nucleic Acid Kit (QIAGEN Cat. no. 55114) according to the manufacturer’s instructions. Final carrier RNA concentration of 1.3 ng/μL was used to improve yields. For quality control, plasma DNA fragment size and concentrations were measured using high sensitivity (HS) Bioanalyzer chips or TapeStation HS D1000 tape cartridges. The plasma DNA concentration and size range that was measured for QC was gated on fragments within the 100–700-bp range to exclude contaminating high molecular weight genomic DNA when present.

### DNA isolation and sequencing of tissue samples

PBMC layer or plasma pellets were utilized to extract the reference genomic DNA using QIAGEN Kits (Cat. no. 51106 and 56304, respectively) according to the manufacturer’s instructions. FFPE DNA from metastatic tissues was isolated using the QIAamp DNA FFPE Tissue Kit (QIAGEN Cat. no. 56404) following the manufacturer’s instructions.

The isolated DNA quality was assayed through agarose gel electrophoresis or TapeStation genomic DNA tapes to determine size and concentration. The resulting gDNA was used to construct low-input DNA sequencing libraries with the Hyper Prep kit (Kapa Biosciences cat. no. KK8504) according to the manufacturer’s instructions. Genomic DNA from PBMCs was sonicated to 200 bp (Covaris Peak power 175, Duty Factor 10%, cycles/burst 200, time 180 s, temp 4–7 °C) prior to end repair and a-tailing steps. Post-ligation cleanup was performed with 0.8X AMPure XP beads. PCR amplification of plasma DNA samples was performed at 11 PCR cycles. Final sequencing libraries were split into two pools for either copy number sparse WGS or exome capture. The copy number libraries were sequenced at 36 or 76 cycles single-end on the Illumina HiSeq4000 system. The exome libraries were further captured using the SeqCap EZ Exome V2 kit following the manufacturer’s instructions (Nimblegen-Roche Cat. no. 05860482001). The final exome libraries were sequenced at 76 or 100 paired-end reads on the Illumina HiSeq4000 system.

### Plasma DNA library construction and exome sequencing

The plasma DNA that passed QC by having ≥ 2 ng total DNA and size distributions < 1000 bp were used to construct low-input DNA sequencing libraries with the Hyper Prep kit (Kapa Biosciences cat. no. KK8504) according to the manufacturer’s instructions. Post-ligation cleanup was performed with 0.8X AMPure XP beads. PCR amplification of plasma DNA samples was performed at 11 PCR cycles. Final sequencing libraries were split into two pools for either copy number sparse WGS or exome capture. The copy number libraries were sequenced directly at 36 or 76 cycles single-end on the Illumina HiSeq 4000 system. The exome libraries were captured using the SeqCap EZ Exome V2 kit following the manufacturer’s instructions (Nimblegen-Roche Cat. no. 05860482001). The final exome libraries were sequenced at 76 or 100 paired-end reads on the Illumina HiSeq4000 system.

### Analysis of genomic copy number data using circular binary segmentation (CBS) from plasma DNA and tissues

Reads sequenced were demultiplexed using the “bcltofastq” software (Illumina) and split into individual FASTQ files, allowing a 1 basepair mismatch for barcode edit distance. Copy number profiles were detected from read depth counting of the sequencing data using the “variable binning” pipeline as previously described [47]. This pipeline involved mapping FASTQ files to the human genome assembly NCBI Build 37 (hg19/NCBI37) using Bowtie2 (2.1.0) alignment software [48]. The aligned reads in SAM files were converted to BAM files and sorted using SAMtools (0.1.16) [49]. PCR duplicates were marked and removed using Picard in GATK [50]. The reads were counted using variable bin sizes at an average genomic resolution of 220 kb. Unique normalized read counts were segmented using the CBS [51] method from R Bioconductor “DNAcopy” package followed by MergeLevels [52] to join adjacent segments with non-significant differences in segmented ratios. The parameters used for CBS segmentation were alpha = 0.0001 and undo.prune = 0.05. Default parameters were used for MergeLevels, which removed erroneous chromosome breakpoints. Finally, we used ggplot packages in R to plot the segmentation and log2(ratio) values and annotate prostate cancer genes in regions of amplification and deletion. The prostate cancer gene list of 100 genes was compiled from two published papers [33, 53].

### Calculation of CNA lengths and correlation between plasma and metastatic samples

The neutral copy number state was defined as the median segmentation ratio of all genomic bins. Any group of consecutive bins with the same segmentation ratio not equal to the copy neutral state within a chromosome was defined as a CNA. The CNA length was defined as a difference between the bin start position and bin end position. Every CNA contained a list of bins with the same segmentation ratio values. These segmentation values from the plasma and metastatic samples were used to calculate the correlation values using the Pearson correlation coefficient.

### Detection of mutations in plasma exome sequencing data

The plasma DNA data from each patient was aligned to the human genome reference assembly (hg19) using Bowtie2 (2.1.0) [48] and converted into a binary format (BAM) with Samtools [49]. The SAM file was sorted using samtools, and PCR duplicates were marked using Picard tools [54]. Genome Analysis Toolkit [50] (GATK)’s BaseRecalibrator and PrintReads were used to obtain BAM files that have been recalibrated for base quality scores. Somatic variants were identified using the plasma and matched PBMC samples using GATK’s MuTect2 [55] to generate a variant call format (VCF) file. The filtering functionality of the MuTect2 VCF file was enabled using GATK’s FilterMutectCalls, and the somatic variants classified as “PASS” (high confidence) and “germline risk” (borderline somatic) were retained. The filtered somatic variants were split into single nucleotide variants (SNVs) and indels using GATK’s SelectVariants function. The chromosome number and position of the variants were extracted, and samtools was used to obtain the read counts across all the variant sites. Variants with less than 0.07 variant allele frequency in the plasma sample were removed from analysis. Additionally, variants were removed from analysis if they had more than 2 variant reads in the PBMC samples sequenced at less than 100X depth or more than 0.01 variant allele frequency for samples sequenced at greater than 100X depth. These read depth filters remove the mutect2 “germline risk” calls that are like to be germline mutations and are not somatic mutations. The resulting data was annotated using ANNOVAR [56] by integrating multiple databases, including dbSNP, COSMIC [57], and the Cancer Gene Census [58]. To identify mutations and copy number variations (CNV) in genes of interest, an exon coordinate file was intersected with BedTools [59]. The functional significance of each SNV was predicted using SIFT [60] and POLYPHEN [61]. Mutations with < 0.05 SIFT scores and > 0.85 POLYPHEN scores were considered significant for impacting gene function.

### Identification of concordant mutations in matched metastases

The total number of CNAs per sample was estimated above a baseline threshold for amplifications and deletions in each patient. The CNA correlation between the two sample sources was calculated using the Pearson correlation coefficient of the segmentation means, while the mutational concordance was calculated as the percentage of the ratio between the concordant mutations and the total number of mutations in the plasma and tissue sample combined.

### Survival analysis by plasma DNA concentration

We utilized survival data from 140 baseline CRPC plasma samples who were part of trial NCT01505868 for survival analysis. The samples were categorized into 2 groups based on total cfDNA (> 2 ng or < 2 ng) to prepare libraries. The overall and progression-free survival months and the group information were used for survival analysis using the Kaplan-Meier estimator using the “survminer” library in R (3.5.0). The *p* value of the log-rank test was calculated, and the two groups were considered significantly different if the *p* value was *p* < 0.05.

### Estimating clonal frequencies from mutation data

Non-synonymous and synonymous somatic mutations were identified in the plasma samples for 12 PC patients with 2 to 6 longitudinal samples. The plasma DNA genomic copy number profiles were estimated from the paired-end exome sequencing depth using the R package “ExomeCNV” [62]. For ExomeCNV analysis, the minimum sensitivity and specificity was set to 0.9999 while it was optimized using the AUC criteria. Tumor purity was estimated with THetA2 [63] using the tumor and normal GATK recalibrated bam files while the minimum fraction of the genome with a potential copy number event for the sample was set to 0. The variant allele frequencies from each point mutation were normalized with both exome-derived copy number profiles and estimated tumor purities using PyClone2 (v0.12.9) [35]. The copy number and purity-adjusted clonal frequencies were then used as input for CITUP [36] for the joint calculation and estimation of clonal subpopulations using the optimal trees across the longitudinal timepoints from same patient. Finally, the clonal lineages were plotted with “timescape” [64] using the CITUP tree structures and the clonal frequencies across the longitudinal timepoints for individual PC patients.

### Survival analysis of resistant genes from TCGA

Selected prostate cancer studies [65–79] including the prostate cancer (MSK, 2019), prostate adenocarcinoma (TCGA, provisional), and The Metastatic Prostate Cancer Project that were available on cBioPortal (www.cbioportal.org) were used for survival analysis to determine if genomic aberrations in significant genes associated with resistance were associated with poor patient survival in the expanded patient cohorts. All the genes reported in Additional file 1: Table S3 are tested, and *p* values from log-rank test were calculated. The *p* values from the log-rank test were then adjusted using the Benjamini-Hochberg procedure.

### Cox regression model for OS and PFS using available clinical and genomic parameters

Using data from 70 prostate cancer patients with available clinical data across all clinical and genomic parameters, we performed a Cox regression model using the “survival” R package. We used the coxph function by utilizing one or all the factors as predictors and the survival months as the outcome variable for the univariate or multivariate analysis. Any predictor that had a *p* value < 0.05 was considered statistically significant. For the discrete clinical and genomic parameters, total cfDNA < 2 ng, diploid cfDNA profiles, low disease volume, protracted rate of progression, and prostate disease were used as a reference for the Cox regression models.

## Supplementary information

**Additional file 1.** Fig. S1-S6 and Table S1-S3.

**Additional file 2.** Review History.

## Data Availability

The datasets supporting the conclusions of the article are available under bioproject ID: PRJNA554329 [80].
