# Supplementary material for: Decoding the evolutionary response to prostate cancer therapy by plasma genome sequencing
Source: Genome Biol. 2020 Jul 6;21:162. doi: 10.1186/s13059-020-02045-9 (PMC7336456; doi:10.1186/s13059-020-02045-9)
Supplement: Supplementary file 1 — Additional file 1. Fig. S1-S6 and Table S1-S3. [file 13059_2020_2045_MOESM1_ESM.pdf]

Ramesh et al.
Supplementary Materials

Table of contents

Supplementary Figures

Fig S1. Size distributions of cfDNA in multiple nucleosomes from representative patients ……1

Fig. S2. Correlation of plasma DNA parameters with the clinical features ……………….…….2

Fig. S3. Additional Patients with Single Timepoint Plasma Samples ……………………...….3

Fig. S4. Concordance of cfDNA and metastatic tissue samples in additional patients…………...4

Fig. S5. Genomic Response in Longitudinal cfDNA Samples from Additional Patients………...5

Fig. S6. Purity and Copy Number Normalization of Mutation Frequencies in Longitudinal cfDNA……………………………………………………………………………………..6

Supplementary Tables

Table S1. Cox model for overall and progression-free survival…………...…….…………………7

Table S2. Clinical information on the profiled prostate cancer patients.………………….……9

Table S3. Mutations in Resistance-Associated Clones that Expanded in Response to Therapy...10

Fig. S1- Size distributions of cfDNA in multiple nucleosomes from representative patients. Tape station traces of cfDNA size distributions in 4 representative prostate cancer patients. (a) A patient with no cfDNA peak identified in the plasma, (b) a patient with a single nucleosome peak with a mean size of 146bp, (c) a patient with double nucleosome peaks identified at ~146bp and ~292bp, and (d) a patient with triple nucleosome peaks identified at ~146bp, ~292bp and ~438bp. (e) Distribution of cfDNA concentration (ng/ml) after purification of 4 samples with 4 experimental replicates, with the mean (solid circles) and standard deviation indicated.

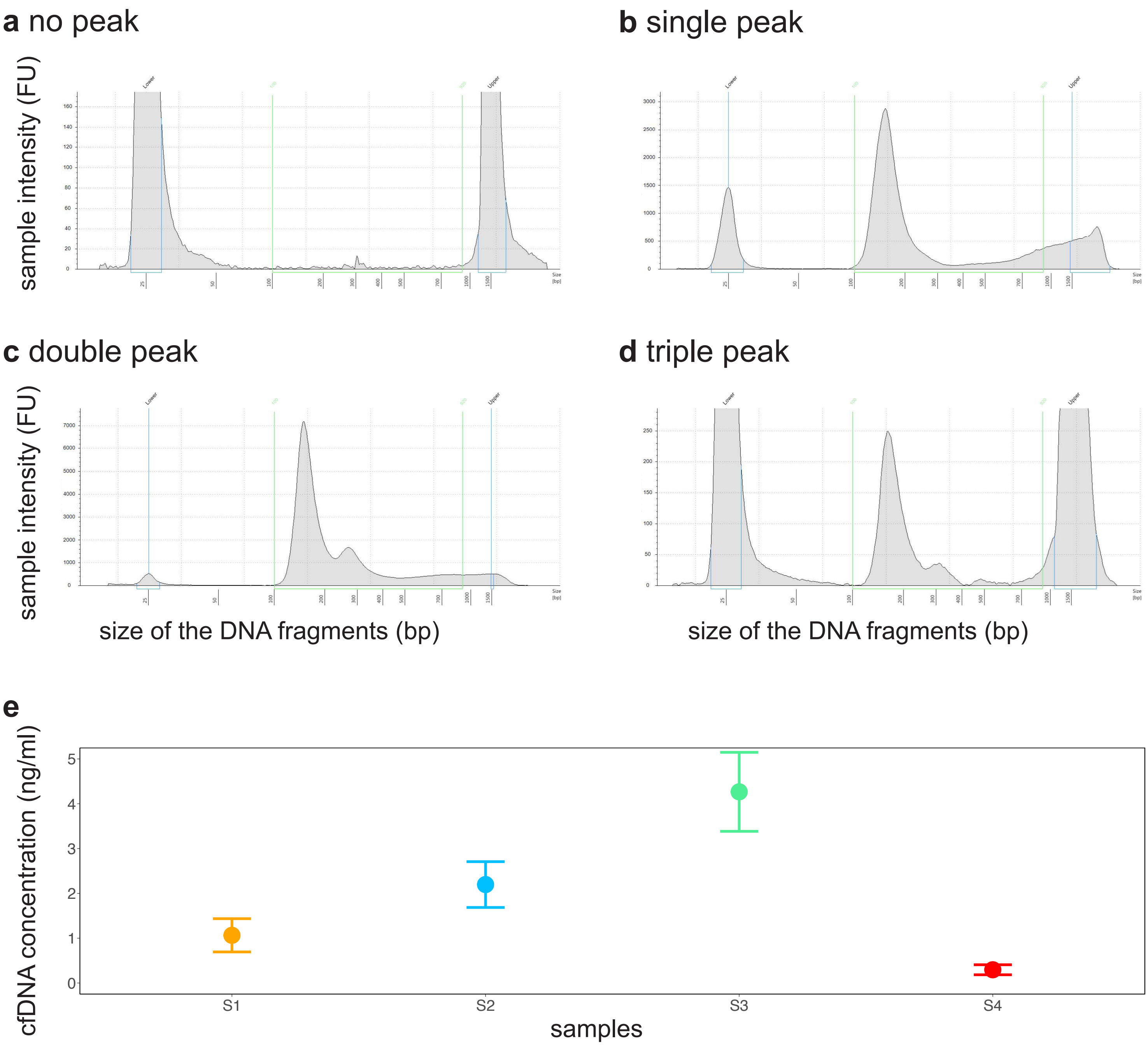

Fig. S2 - Correlation of plasma DNA parameters with clinical features

(a) Comparison of cfDNA concentrations between CSPC and CRPC patients. (b) Kaplan-Meier curve of progression-free survival for patients with total plasma DNA <2 ng and total plasma DNA ≥2 ng. (c) Kaplan-Meier progression-free survival plot for patients with aneuploid and diploid cfDNA CNA profiles. (d) Comparison of the distributions of cfDNA concentrations (ng/mL) between patients with low (GS=6-7) and high grade (GS=8-10) as defined by Gleason scores. (e) Comparison of the distributions of cfDNA concentration (ng/mL) between patients with active disease in the primary site (prostate), lymph node (lym), bone (oss) and/or visceral metastasis (f) cfDNA concentrations (ng/mL) compared to PSA values (ng/mL), with correlation values calculated by Pearson correlation coefficient. Significance for the survival analysis in panels (b,c) was calculated with the log-rank test, while the signficance of the plots in panels (a,d,e) was calculated using the Wilcoxon rank sum test. Red dots in panels (a,d,e) represent mean values.

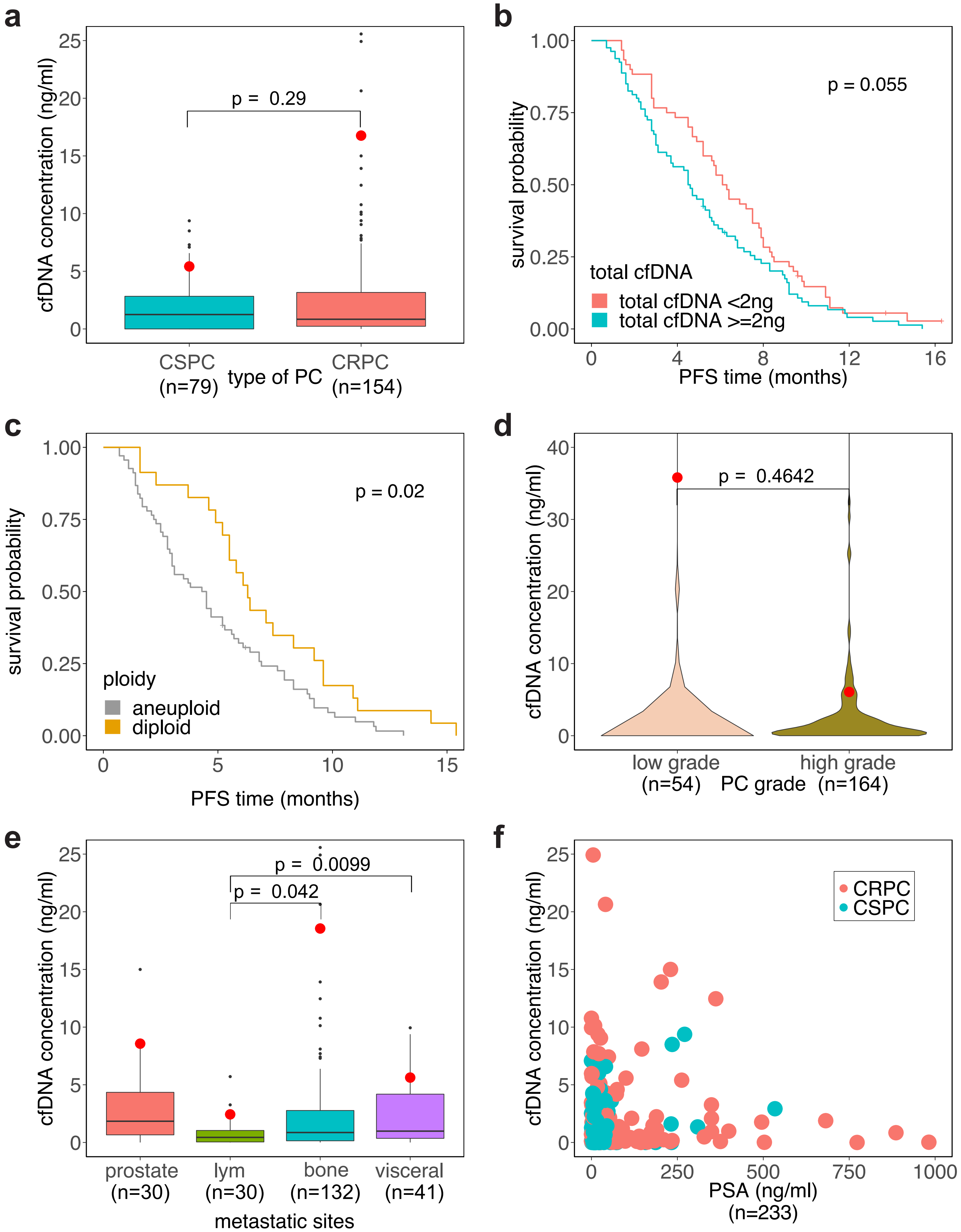

Fig. S3 – Additional Patients with Single Timepoint Plasma Samples

(a) Genomic copy number ratio and segmentation plots of cfDNA from 4 prostate cancer patients, with annotations of prostate cancer genes amplified in red boxes and lost in blue boxes. (b) Circos plots of CNAs, indels and point mutations for the plasma DNA of the 4 patients with annotations of prostate cancer genes labeled in the outer ring. (c) Box plot representing the fraction of genome with CNAs. The copy neutral state has been set to the median segmentation value. (d) Distribution of the CNA lengths (kb). The copy neutral state has been defined as the median segmentation value.

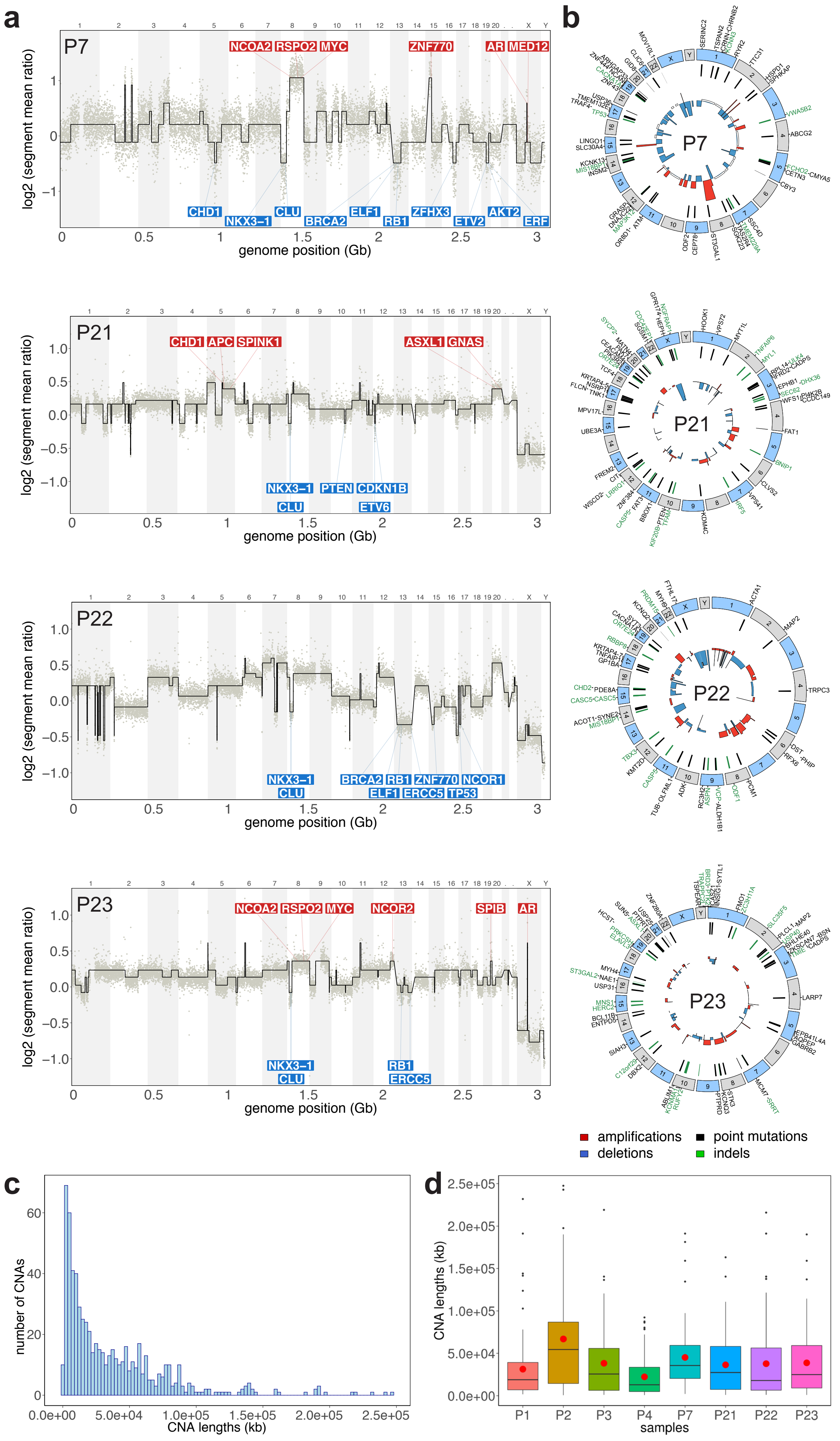

Fig. S4 - Concordance of cfDNA and metastatic tissue samples in additional patients.
(a-f) Genomic copy number data and exome mutations for 6 additional PC patients with matched metastatic tissue samples, with prostate cancer genes labeled.

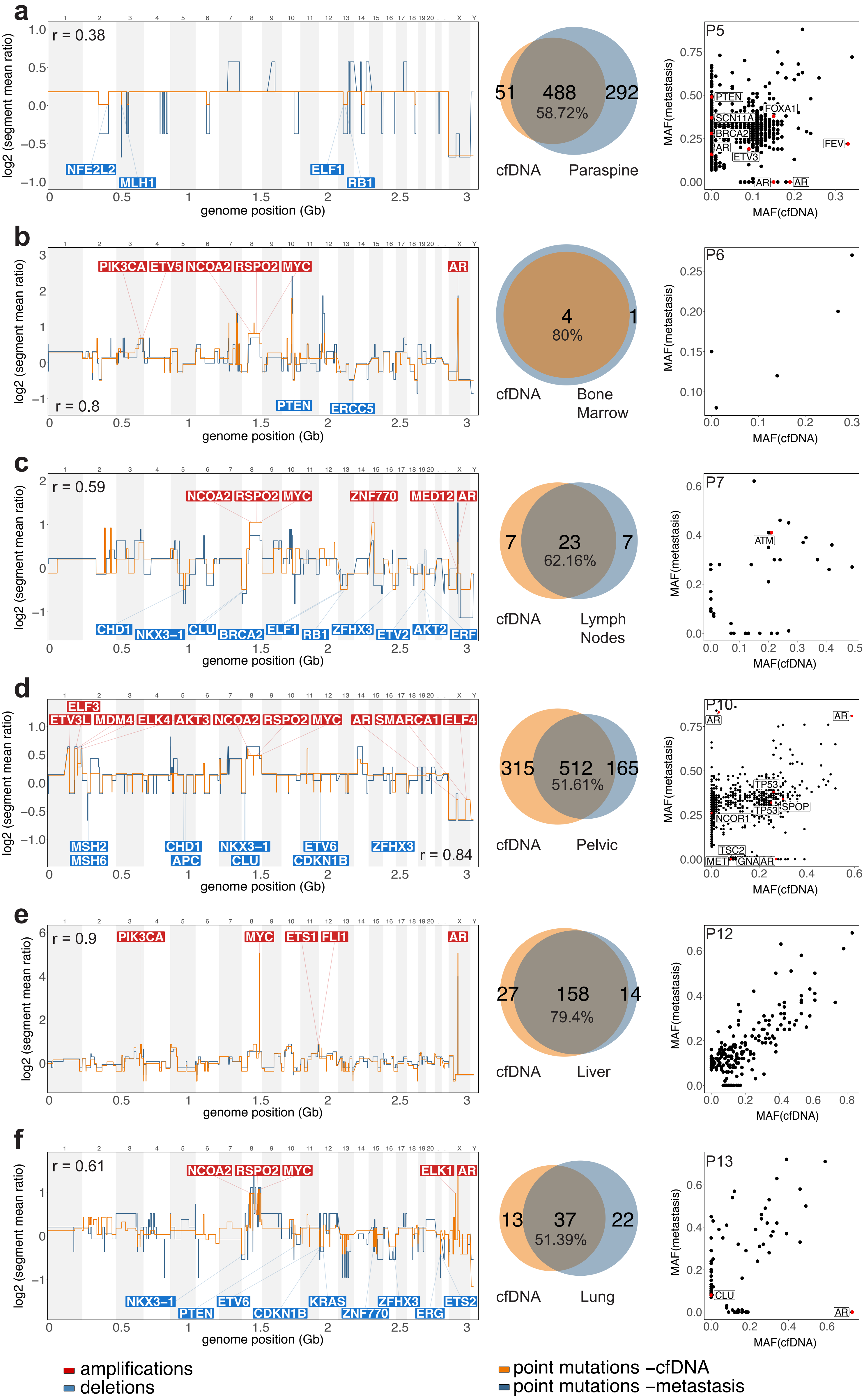

Fig. S5 – Genomic Response in Longitudinal cfDNA Samples from Additional Patients.
(a-f) Plots of treatment schedules and therapeutic agents against changes in PSA levels (ng/mL) in 6 additional PC patients, with genomic copy number heatmaps and exome MAF plotted below for each time point. Colors in mutation line plots represent different clones inferred by CITUP (methods), while blue colors in PSA plots represent time points that were sampled for sequencing analysis.

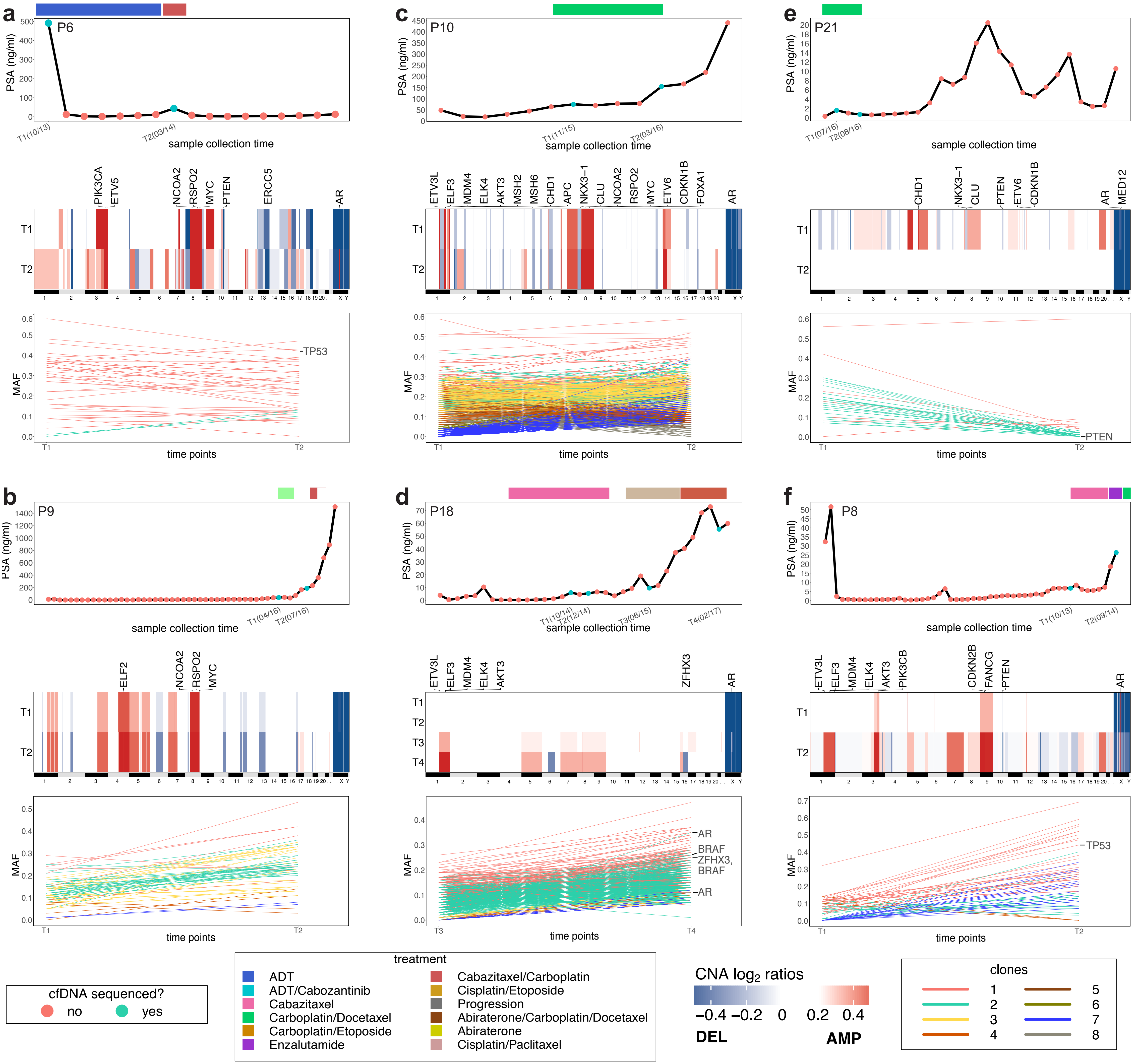

Fig. S6 – Purity and Copy Number Normalization of MAF in Longitudinal cfDNA Data.
(a-l) Left panels showing raw exome MAF prior to purity correction and copy number normalization compared to right panels showing the inferred clonal frequencies after normalization for the 12 prostate cancer patients.

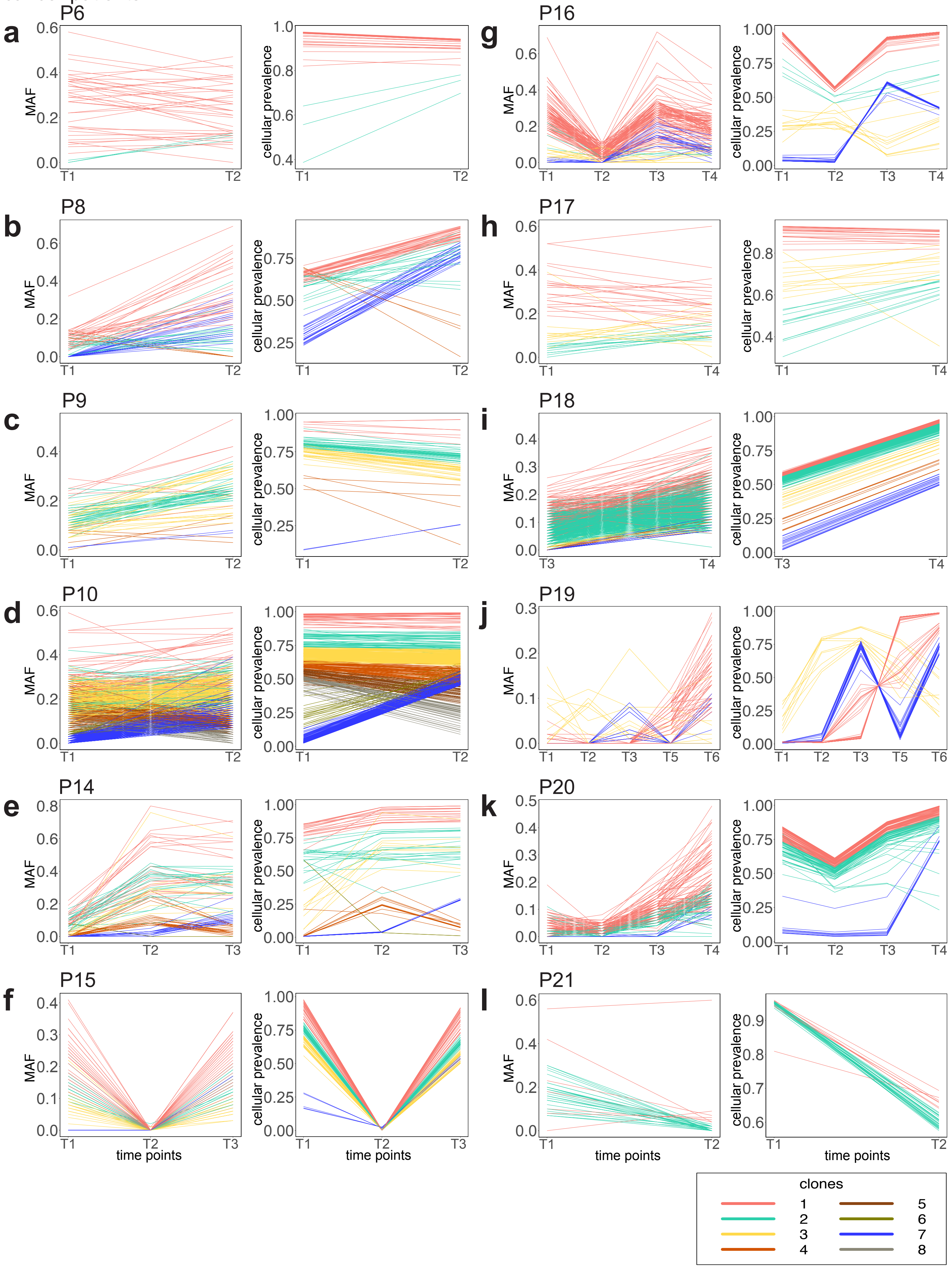

Table S1 – Cox proportional hazards regression model (a) Overall and (b) Progression-Free Survival Analysis using clinical and genomic factors as predictors with (1) total cfDNA as a predictor, (2) ploidy of the cfDNA as a predictor, (3) total cfDNA and ploidy of cfDNA as predictors, (4) total cfDNA, ploidy of cfDNA and its interaction as predictors, (5) total cfDNA, ploidy of cfDNA, cfDNA fragment size, disease volume and type of progression.

a. Overall Survival (OS)

n= 70, number of events= 53

1. OS (outcome); total cfDNA (< or >= 2ng) (predictor)

predictors	coef	exp(coef)	se(coef)	lower 0.95	upper 0.95	z	Pr(>|z|)
total_cfDNA_>=2ng	0.5772	1.781	0.4374	0.7556	4.198	1.319	0.187

Concordance= 0.55 (se = 0.026)
Likelihood ratio test= 2 on 1 df, p=0.2
Wald test = 1.74 on 1 df, p=0.2
Score (logrank) test = 1.79 on 1 df, p=0.2

2. OS (outcome); ploidy of the cfDNA (Diploid vs Aneuploid) (predictor)

predictors	coef	exp(coef)	se(coef)	lower 0.95	upper 0.95	z	Pr(>|z|)	signifiance
Aneuploid	0.8009	2.2275	0.3407	1.142	4.344	2.351	0.0187	*

Concordance= 0.594 (se = 0.031)
Likelihood ratio test= 6.34 on 1 df, p=0.01
Wald test = 5.53 on 1 df, p=0.02
Score (logrank) test = 5.82 on 1 df, p=0.02

3. OS (outcome); total cfDNA (< or >= 2ng) and ploidy of the cfDNA (Diploid vs Aneuploid) (predictors)

predictors	coef	exp(coef)	se(coef)	lower 0.95	upper 0.95	z	Pr(>|z|)	signifiance
total_cfDNA_>=2ng	0.407	1.5023	0.4434	0.63	3.583	0.918	0.3587	
Aneuploid	0.7435	2.1033	0.345	1.07	4.136	2.155	0.0312	*

Concordance= 0.614 (se = 0.034)
Likelihood ratio test= 7.27 on 2 df, p=0.03
Wald test = 6.27 on 2 df, p=0.04
Score (logrank) test = 6.59 on 2 df, p=0.04

4. OS (outcome); total cfDNA (< or >= 2ng), ploidy of the cfDNA (Diploid vs Aneuploid) and interaction of total cfDNA and ploidy of cfDNA (predictors)

predictors	coef	exp(coef)	se(coef)	lower 0.95	upper 0.95	z	Pr(>|z|)
total_cfDNA_>=2ng	0.5842	1.7935	0.7851	0.3849	8.356	0.744	0.457
Aneuploid	0.966	2.6273	0.8689	0.4785	14.426	1.112	0.266
total_cfDNA_>=2ng*Aneuplo	-0.2651	0.7671	0.9445	0.1205	4.885	-0.281	0.779

Concordance= 0.614 (se = 0.034)
Likelihood ratio test= 7.35 on 3 df, p=0.06
Wald test = 6.12 on 3 df, p=0.1
Score (logrank) test = 6.59 on 3 df, p=0.09

5. OS (outcome); total cfDNA (< or >= 2ng), ploidy of the cfDNA (Diploid vs Aneuploid), cfDNA fragment size, disease volume (low vs intermediate and high), type of progression (protracted vs accelerated) and metastatic sites (none/prostate vs lymph node, bones +/- lymph nodes and visceral+) (predictors)

predictors	coef	exp(coef)	se(coef)	lower 0.95	upper 0.95	z	Pr(>|z|)	signifiance
total_cfDNA_>=2ng	0.409977	1.506784	0.457258	0.61494	3.692	0.897	0.3699	
Aneuploid	0.857022	2.356133	0.40298	1.0695	5.191	2.127	0.0334	*
cfDNA_fragment_size	0.01183	1.0119	0.010633	0.99103	1.033	1.113	0.2659	
disease_volume_intermediat	0.2379	1.268583	0.856011	0.23696	6.791	0.278	0.7811	
disease_volume_high	0.271155	1.311478	0.833771	0.25589	6.722	0.325	0.745	
progression_accelerated	0.150931	1.162917	0.355396	0.57948	2.334	0.425	0.6711	
grade_high	NA	NA	0	NA	NA	NA	NA	
LN	0.332071	1.393852	0.810142	0.28485	6.82	0.41	0.6819	
Bones+/-LN	-0.017465	0.982687	0.746171	0.22765	4.242	-0.023	0.9813	
Visceral+	-0.827227	0.43726	0.7982	0.09148	2.09	-1.036	0.3	
PSA	0.000376	1.000376	0.00025	0.99989	1.001	1.504	0.1326	

Concordance= 0.667 (se = 0.039)
Likelihood ratio test= 16.69 on 10 df, p=0.08

Wald test = 15.45 on 10 df, p=0.1
Score (logrank) test = 16.47 on 10 df, p=0.09

b. Progression-Free Survival (PFS)

n= 70, number of events= 69

1. PFS (outcome); total cfDNA (< or >= 2ng) (predictor)

predictors	coef	exp(coef)	se(coef)	lower 0.95	upper 0.95	z	Pr(>|z|)
total_cfDNA_>=2ng	0.2171	1.2425	0.3321	0.648	2.382	0.654	0.513

Concordance= 0.528 (se = 0.027)
Likelihood ratio test= 0.45 on 1 df, p=0.5
Wald test = 0.43 on 1 df, p=0.5
Score (logrank) test = 0.43 on 1 df, p=0.5

2. PFS (outcome); ploidy of the cfDNA (Diploid vs Aneuploid) (predictor)

predictors	coef	exp(coef)	se(coef)	lower 0.95	upper 0.95	z	Pr(>|z|)
Aneuploid	0.4749	1.6079	0.2767	0.9348	2.766	1.716	0.0861

Concordance= 0.572 (se = 0.029)
Likelihood ratio test= 3.15 on 1 df, p=0.08
Wald test = 2.95 on 1 df, p=0.09
Score (logrank) test = 3 on 1 df, p=0.08

3. PFS (outcome); total cfDNA (< or >= 2ng) and ploidy of the cfDNA (Diploid vs Aneuploid) (predictors)

predictors	coef	exp(coef)	se(coef)	lower 0.95	upper 0.95	z	Pr(>|z|)
total_cfDNA_>=2ng	0.02602	1.02636	0.35158	0.5153	2.044	0.074	0.941
Aneuploid	0.4677	1.59631	0.2933	0.8984	2.836	1.595	0.111

Concordance= 0.576 (se = 0.034)
Likelihood ratio test= 3.15 on 2 df, p=0.2
Wald test = 2.95 on 2 df, p=0.2
Score (logrank) test = 3 on 2 df, p=0.2

4. PFS (outcome); total cfDNA (< or >= 2ng), ploidy of the cfDNA (Diploid vs Aneuploid) and interaction of total cfDNA and ploidy of cfDNA (predictors)

predictors	coef	exp(coef)	se(coef)	lower 0.95	upper 0.95	z	Pr(>|z|)
total_cfDNA_>=2ng	0.4437	1.5585	0.5315	0.5499	4.417	0.835	0.4038
Aneuploid	1.0711	2.9186	0.6161	0.8725	9.763	1.739	0.0821
total_cfDNA_>=2ng*Aneuplo	-0.7713	0.4624	0.6936	0.1187	1.801	-1.112	0.2662

Concordance= 0.586 (se = 0.033)
Likelihood ratio test= 4.38 on 3 df, p=0.2
Wald test = 4 on 3 df, p=0.3
Score (logrank) test = 4.15 on 3 df, p=0.2

5. PFS (outcome); total cfDNA (< or >= 2ng), ploidy of the cfDNA (Diploid vs Aneuploid), cfDNA fragment size, disease volume (low vs intermediate and high), type of progression (protracted vs accelerated) and metastatic sites (none/prostate vs lymph node, bones +/- lymph nodes and visceral+) (predictors)

predictors	coef	exp(coef)	se(coef)	lower 0.95	upper 0.95	z	Pr(>|z|)	signifiance
total_cfDNA_>=2ng	0.119201	1.126597	0.376241	0.5389	2.355	0.317	0.7514	
Aneuploid	0.794438	2.213197	0.316764	1.1896	4.118	2.508	0.0121	*
cfDNA_fragment_size	0.009014	1.009055	0.009448	0.9905	1.028	0.954	0.34	
disease_volume_intermediat	-0.001088	0.998913	0.870019	0.1815	5.497	-0.001	0.999	
disease_volume_high	-0.327994	0.720367	0.889385	0.126	4.117	-0.369	0.7123	
progression_accelerated	0.085318	1.089064	0.300115	0.6048	1.961	0.284	0.7762	
grade_high	NA	NA	0	NA	NA	NA	NA	
LN	0.793291	2.21066	0.809597	0.4523	10.806	0.98	0.3272	
Bones+/-LN	1.090723	2.976425	0.75464	0.6782	13.063	1.445	0.1484	
Visceral+	0.797612	2.220233	0.772719	0.4883	10.096	1.032	0.302	
PSA	-0.00028	0.999721	0.000316	0.9991	1	-0.884	0.3767	

Concordance= 0.611 (se = 0.038)
Likelihood ratio test= 10.8 on 10 df, p=0.4
Wald test = 10.39 on 10 df, p=0.4
Score (logrank) test = 10.7 on 10 df, p=0.4

Table S2 – Clinical information on the profiled prostate cancer patients. Clinical information and treatments administered to the metastatic CSPC and CRPC patients that were analyzed using PEGASUS in this study. Columns listed include clinical parameters for: (1) the patient identifier, (2) date of first plasma sample collection analyzed (3) classification of castration-sensitive or resistant disease at the data of sample collection, (4) patient age, (5) prostate specific antigen levels (ng/mL), (6) Gleason Score, (7) site of metastatic tumors, (8) progression-free survival in days, (9) overall survival in days, (10) treatments received starting at the date of the first plasma sample collection, and (11) prior treatment received by the patient before the first plasma sample collection.

patient	date	prostate cancer subtype	age	PSA	GS	Metastatic Sites	PFS (days)	OS (days)	on-study treatment	previous treatments
P1	9/2/15	CRPC	61	0.4	9 (4+5)	Lymph Nodes, Bones	171	262	Docetaxel + Carboplatin	Abiraterone+Enzalutamide, Cabazitaxel+Carboplatin
P2	4/25/14	CRPC	64	1.5	9 (4+5)	Lymph Nodes	276	828	Cabazitaxel	ADT, Radiation
P3	12/18/13	CRPC	63	5	9 (4+5)	Bones, Liver	42	207	Cabazitaxel	Radiation ,Bicalutamide, Abiraterone+Enzalutamide
P4	05/23/16	CRPC	74	234.8	9 (4+5)	Lymph Nodes, Bones, Lung	87	189	Docetaxel + Carboplatin	Cabazitaxel+Carboplatin, Radiation, CVD
P5	7/15/13	CRPC	72	146.5	9 (4+5)	Bones	299	742	Cabazitaxel+Carboplatin	Bicalutadmide, Nilut, Abiraterone
P6	10/22/13	CSPC	61	491.5	9 (4+5)	Lymph Nodes, Bones	161	1061	ADT	Naïve
P7	2/16/15	CRPC	75	91.9	9 (4+5)	Lymph Nodes, Bones	186	243	Cabazitaxel+Carboplatin	ADT, Bicalutamide
P8	10/24/13	CRPC	69	6.8	9 (4+5)	Lymph Nodes, Bones	133	522	Cabazitaxel	Bicalutamid, Abiraterone, Dasatinib, Sunitinib
P9	4/14/16	CRPC	67	39.6	9 (4+5)	Lymph Nodes, Bones, Adrenal	50	205	Docetaxel	ADT, Abiraterone+Sunitinib
P10	3/28/16	CRPC	55	154.8	8 (4+4)	Lymph Nodes, Bones	68	141	Cabazitaxel + Carboplatin + Abiraterone	ADT+Cabozantinib, Docetaxel+Carboplatin, Cabazitaxel+Carboplatin
P11	5/16/16	CRPC	67	231.8	10 (5+5)	None	289	NR	ADT	Naïve
P12	5/23/16	CRPC	69	31.7	8 (4+4)	Bones, Liver	75	106	Carboplatin + Etoposide	Radiation, Radium223, Docetaxel+Carboplatin, Enzalutamide, CVD, Abiraterone, Cabazitaxel, Docetaxel
P13	10/14/16	CRPC	68	349.3	7 (4+3)	Lymph Nodes, Bones	60	90	Cyclophosphamide	bicalutamide, Enzalutamide, XRT
P14	2/27/14	CRPC	59	5603.4	NA	Lymph Nodes, Bones, Lung	102	561	ADT	Naïve
P15	4/15/14	CRPC	73	79.7	7 (3+4)	Lymph Nodes, Bones	302	509	Cabazitaxel	Bicalutamide, Enzalutamide
P16	5/19/14	CRPC	77	72.6	6 (3+3)	Lymph Nodes, Bones	136	519	Cabazitaxel	Bicalutamide
P17	1/20/15	CRPC	59	27.7	8 (4+4)	Lymph Nodes, Bones,Liver	84	269	Cabazitaxel+Carboplatin	ADT, Enzalutamide
P18	10/17/14	CRPC	73	6	9 (4+5)	Bones, Adrenal Gland	208	694	Cabazitaxel	ADT, Bicalutamide, Abiiraterone+Enzalutamide
P19	8/6/14	CSPC	62	12.9	9 (5+4)	Lymph Nodes, Bones	171	533	ADT + Cabozanitib	Naïve (T<20)
P20	6/12/15	CSPC	75	1.9	9 (5+4)	Bones	180	883	ADT + Cabozantinib	Naïve (3 mos on ADT)
P21	7/7/16	CRPC	67	1.6	9 (4+5)	Lymph Nodes, Bones	213	NR	Docetaxel + Carboplatin	Bicalutamide
P22	01/05/16	CSPC	60	234.9	9 (4+5)	Lymph Nodes, Bones	150	484	ADT	naïve
P23	06/17/16	CRPC	61	171.1	9 (5+4)	Bones, Lung, Liver	277	575	Cabazitaxel+Carboplatin	Docetaxel, Sipuleucel-T, Enzalutamide, Abiraterone

Table S3 – Mutations in Resistance-Associated Clones that Expanded in Response to Therapy. List of the significant mutations with SIFT (S< 0.05) and Polyphen (P> 0.85) scores in the resistant clones that expanded in response to therapy in 9 of the prostate cancer patients with serial data. The columns listed include: (1) patient identifier, (2) inferred CITUP clone number (3) gene names, (4) reference nucleotide variant, (5) mutant nucleotide variant, (6) chromosome number, (7) chromosome position, (8) Polyphen2 significance score, (9) SIFT score, (10) mutation type, including nonsynonymous (NSN) or stopgain (STOP), (11-15) raw variant allele frequencies of the somatic mutations in the plasma at different time points (16) GenBank identifier, (17) short description of the gene function, (18) Overall survival p-value for genes in TCGA datasets, (19) Benjamini-hochberg adjusted p-values for genes in TCGA datasets.

patient	cloneid	gene	Ref	Mut	chrom	position	POLY	SIFT	type	T1_VAF	T2_VAF	T3_VAF	T4_VAF	T5_VAF	GenBank ID	Function	p-value (OS)	adj p-value (OS)
P20	3	CAPN2	C	T	chr1	223936795	0.998	0	NSN	0	0	0	0.1	0	NM_001146068	cystein protease	0.005874	0.028
P20	3	MRPL47	G	A	chr3	179316479	1	0.01	NSN	0	0	0.01	0.07	0	NM_020409	mitochondrial ribosomal protein	0.121	0.19
P8	3	C8orf48	T	C	chr8	13425294	1	0	NSN	0	0.3	0	0		NM_001007090	Chromosome 8 Open Reading Frame 48	0.0513	0.107
P8	3	CDHR2	C	T	chr5	176017681	0.999	0	NSN	0	0.17	0.02	0		NM_001171976	calcium-dependent cell-cell adhesion molecules	0.000002986	0
P8	3	FAM196A/INSYN2A	G	A	chr10	128974496	1	0.02	NSN	0	0.22	0	0		NM_001039762	inhibitory synaptic factor 1A	0.6	0.683
P8	3	LGALS3	G	A	chr14	55604883	1	0.01	NSN	0	0.26	0	0		NM_001177388	apoptosis, innate immunity, cell adhesion and T-cell regulation	0.0005425	0.004
P8	3	SLC12A3	A	T	chr16	56914156	0.996	0	NSN	0	0.29	0	0		NM_001126108	electrolyte homeostasis	0.246	0.337
P8	3	POSTN	T	A	chr13	38160330	NA	1	STOP	0	0.22	0	0		NM_001286665	tissue development and regeneration, including wound healing	0.699	0.744
P6	2	SLC38A7	C	T	chr16	58713853	0.99	NA	NSN	0	0.12	0	0		NM_018231	L-glutamate and L-serine transmembrane transporter	0.426	0.521
P14	5	DSCAM	A	C	chr21	42064766	0.999	0.01	NSN	0	0.01	0.11	0		NM_001389	human central and peripheral nervous system development	0.0446	0.107
P14	5	EEF1A2	C	T	chr20	62126325	0.999	0	NSN	0.01	0.02	0.1	0		NM_001958	enzymatic delivery of aminoacyl tRNAs to the ribosome	0.0501	0.107
P14	5	ITGAX	T	C	chr16	31372385	1	0	NSN	0	0.01	0.1	0		NM_001286375	adherence of neutrophils and monocytes to endothelium	1	1
P14	5	L1CAM	C	A	chrX	153134182	1	0	NSN	0	0.05	0.24	0		NM_024003	nervous system development, migration and differentiation	0.0005751	0.004
P14	5	MORC1	G	A	chr3	108724041	0.965	0.01	NSN	0	0.01	0.1	0		NM_014429	early spermatogenesis	0.012	0.044
P14	5	WWC1	C	G	chr5	167824751	0.999	0.02	NSN	0	0	0.09	0		NM_015238	enhance memory in some individuals	0.74	0.763
P16	4	EIF3G	T	C	chr19	10226382	0.996	0.01	NSN	0	0.02	0.15	0.08	0	NM_003755	initiation of protein translation	0.000117	0.001
P16	4	MRPL2	T	A	chr6	43025835	1	0.03	NSN	0	0	0.19	0.08	0	NM_001300848	protein synthesis within the mitochondria	0.0924	0.169
P17	3	DYNC1LI2	G	T	chr16	66785226	0.999	0.01	NSN	0.03	0.12	0			NM_006141	microtubule-associated motor protein	0.0737	0.143
P17	3	OR8A1	G	C	chr11	124440816	0.985	0	NSN	0.02	0.09	0			NM_001005194	olfactory receptor 8A1	0.004955	0.027
P17	3	PLCB2	T	C	chr15	40590843	0.891	0.01	NSN	0.02	0.09	0			NM_001284298	regulator of platelet responses	0.212	0.318
P18	5	OR2AE1	G	A	chr7	99474041	0.988	0.03	NSN	0	0.09	0			NM_001005276	olfactory receptor 2AE1	0.0174	0.057
P18	5	SLC18A3	C	A	chr10	50818911	0.997	0	NSN	0	0.08	0			NM_003055	transports acetylcholine vesicles in extracellular space	0.348	0.442
P18	5	TNIK	G	A	chr3	170858205	0.996	0	NSN	0.08	0.06	0			NM_001161565	carcinogenesis and embryonic development	0.00756	0.031
P15	4	FRMD3	A	T	chr9	85926888	1	0.01	NSN	0	0	0.17	0		NM_001244959	primarily found in ovaries but function not clear	0.227	0.326
P15	4	NCAPG	G	T	chr4	17838788	NA	0.02	STOP	0	0	0.07	0		NM_022346	condensation of chromosomes during mitosis and meiosis	0.651	0.716
P19	2	GPR98/ADGRV1	C	A	chr5	90106409	1	NA	NSN	0.01	0	0.01	0	0.1	NM_032119	expressed in the central nervous system	0.0521	0.107
P19	2	MYOM1	C	A	chr18	3086100	0.968	0.05	NSN	0	0	0.01	0	0.07	NM_003803	involved in striated muscle contraction	0.255	0.337
P19	2	SLC26A2	G	T	chr5	149360757	1	0	NSN	0	0	0.02	0	0.07	NM_000112	pathogenesis of several human chondrodysplasias	0.112	0.185
P19	2	SMYD3	C	A	chr1	246670393	1	0.01	NSN	0	0	0.03	0	0.08	NM_001167740	functions in RNA polymerase II by interaction with RNA helicase	1.34E-12	0
P19	2	SYCP2	C	A	chr20	58489301	0.952	0	NSN	0.01	0	0.03	0	0.08	NM_014258	links homologous chromosomes during the prophase of meiosis	0.0241	0.072
P19	2	TMEM151B	G	T	chr6	44243758	0.999	0.01	NSN	0	0	0.08	0	0.03	NM_001137560	transmembrane protein 151b	0.591	0.683
P19	2	ZNF639	G	T	chr3	179051977	1	0.04	NSN	0	0	0.02	0	0.07	NM_016331	may regulate HIV-1 gene expression	0.111	0.185
P19	2	ZNF638	C	A	chr2	71653915	NA	0	STOP	0	0	0.01	0	0.07	NM_001014972	associated with packaging, transferring, or processing transcripts	0.0412	0.107
