# Supplementary material for: Decoding the evolutionary response to prostate cancer therapy by plasma genome sequencing
Source: Genome Biol. 2020 Jul 6;21:162. doi: 10.1186/s13059-020-02045-9 (PMC7336456; doi:10.1186/s13059-020-02045-9)
Supplement: Supplementary file 2 — Additional file 2. Review History. [file 13059_2020_2045_MOESM2_ESM.docx]

Review History

**First round of review**

**Reviewer 1**

**Are you able to assess all statistics in the manuscript, including the appropriateness of statistical tests used?**

Yes, and I have assessed the statistics in my report.

**Comments to author:**

This paper used unbiased genome-wide sequencing of cfDNA to profile copy number variations and exome mutations from archival cryostored plasma samples of prostate cancer patients, in order to infer clonal dynamics and genome evolution in response to treatment.  The authors developed an experimental approach (the PEGASUS method) that takes advantage of the size difference between cfDNA and WBC contamination to enrich cfDNA. They observed associations between cfDNA molecular properties and clinical outcomes, as well as a concordance of mutations between cfDNA and metastatic tumor tissue. By longitudinally tracking the genomic changes during treatment, they identified clones harboring mutations associated with resistance. The paper shall contribute to the longitudinal analysis of cfDNA, for which very few papers have been published. All raw data and clinical information shall be made public, and those should be good resources for the community.  

Major points:
1.     The approach implemented a size selection step to get rid of genomic DNA released from WBC during sample preparation. However, the authors didn't show the enrichment of cfDNA or ctDNA after this size selection step. They should show some data to show the effectiveness of the PEGASUS method.
2.     In line 193, the authors showed the number of CNAs in the single-time-point plasma samples from 8 CRPC patients. Besides the number of CNAs, the length of the genome affected by the CNAs might be more important, as CNAs have varied lengths. The authors should provide statistics about the length of CNAs.
3.     To demonstrate the concordance of CNAs from plasma and metastatic tissue, the authors showed the correlated CNA burden (the number of CNAs). The number of CNAs alone could be too rough to prove the concordance, as the CNAs may have different breakpoints and different lengths. Besides the analysis of CNA burden, the author should compare the genomic position and the span of CNAs between matched plasma-tissue pairs.
4.     Line 506 - the authors said they used the correlation of the segmentation means to calculate the CNA correlation. The segmentation mean may refer to the mean depth of coverage within a segment. If true, then it is possible that the correlation was biased by the unmutated regions. The authors should justify this measurement.

Minor points:
1.     Line 160 - the authors used "mutation frequency", which could refer to both mutation base frequency across the genome or mutation allele frequency. Probably the authors meant mutation allele frequency based on Fig. 1f. The authors should use a more precise word.  
2.     Line 179 -  the authors used "cfDNA quantity", where they might mean "cfDNA concentration" based on Fig. S2e. The authors should use a consistent and precise word.
3.     Line 490 - the authors retained mutations labeled as "germline risk" by GATK's FilterMutect2Calls when they called somatic mutations. The authors should justify the reason to include these mutations.

**Reviewer 2**

**Are you able to assess all statistics in the manuscript, including the appropriateness of statistical tests used?**

Yes, and I have assessed the statistics in my report.

**Comments to author:**

The manuscript by Ramesh et al. described a new approach enrich and profile plasma cfDNA for over 200 prostate cancer patients. They showed the amount of cfDNA is associated with aneuploidy and patient survival. For a small number of patients, genome and exome sequencing were performed to interrogate the CNV and mutation profiles. Furthermore, a subset of patients have longitudinal blood samples which were then used to infer clonal evolution upon treatment. As a non-invasive approach, cfDNA study is certainly of clinical significance and has received much attention in the past few years. This manuscript presented a thorough study of cfDNA in prostate cancer. In general, the data were clear and the manuscript was well-written. There were several places the authors failed to provide enough details or over-interpret the results. The authors have discussed the limitations of their approach, but some of their conclusions were misleading. The details are described below:
1.     The first half of the manuscript focused on the associations between cfDNA level with many other factors. However, how sample processing step contribute to the variation of cfDNA level was not assessed. I wish the authors can extract the cfDNA from the same sample multiple times to quantify the variation between replicates. This would be a critical QC. In addition, when the authors used term cfDNA level, sometimes it referred to total cfDNA, sometimes concentration of cfDNA. What's the difference between these two measurements? If the input amount of blood is the same, these should be the same.
2.     In Figure1 and 2 as well as suppl. figures, the authors presented many associations between various factors and patient survival. Some were significant others were not. It is very hard to fully grasp all the information. It would be better to integrate all the factors into a Cox regression model and resolve the dependency. For example, both cfDNA level and aneuploidy were associated with survival. cfDNA level and aneuploidy were also associated. I wonder in a regression model, would both cfDNA level and aneuploidy carry independent predictive power.
3.     In method, the authors mentioned "Patients were classified as aneuploid or diploid based on the whole genome copy number data from the plasma". It seemed to be a very subjective judgement, not reproducible by other. What are the ploidy of samples shown in Fig 3 and 4?
4.     It is interesting to see that aneuploidy is associated with smaller DNA size. But I can't think of any possible explanation of how this could be.
5.     Based on the CNV and mutation patterns of P15 and P16, the authors used the term "genomic response to treatment". I don't think the observed pattern can be claimed as genomic response. A more likely case would be tumor stopped shedding DNA during that time. Or there might be something wrong with the sample.
6.     Page 12, "In these patients, the increase in genomic aberrations over time was likely due to increasing tumor purity". Why not increased tumor volume, or increased DNA release from tumor?
7.     The authors claimed "persisted through all of the timepoints". However, in P14, aneuploidy seemed to increase through treatment.
Some minor concerns:
1.     Abstract format is not GB style.
2.     Words in title don't need to be listed as key words again.

**Authors Response**

**Point-by-point responses to the reviewers’ comments:**

Reviewer 1 Responses

Reviewer #1: This paper used unbiased genome-wide sequencing of cfDNA to profile copy number variations and exome mutations from archival cryostored plasma samples of prostate cancer patients, in order to infer clonal dynamics and genome evolution in response to treatment. The authors developed an experimental approach (the PEGASUS method) that takes advantage of the size difference between cfDNA and WBC contamination to enrich cfDNA. They observed associations between cfDNA molecular properties and clinical outcomes, as well as a concordance of mutations between cfDNA and metastatic tumor tissue. By longitudinally tracking the genomic changes during treatment, they identified clones harboring mutations associated with resistance. The paper shall contribute to the longitudinal analysis of cfDNA, for which very few papers have been published. All raw data and clinical information shall be made public, and those should be good resources for the community.

Major points:
R1.C1
1. The approach implemented a size selection step to get rid of genomic DNA released from WBC during sample preparation. However, the authors didn't show the enrichment of cfDNA or ctDNA after this size selection step. They should show some data to show the effectiveness of the PEGASUS method.
*Response: We do not expect or claim that the isolation of the cfDNA following the PEGASUS protocol provides an enrichment of the tumor-specific ctDNA fraction over the high-molecular weight DNA from the white blood cells that are also in the plasma during column-purification. However, by centrifuging the WBCs three times, our protocol does remove high-molecular weight DNA that is present in cells that may contaminate the ctDNA. We have clarified this point in the revised manuscript and avoided any use of the word ‘enrichment’ in the main text in regards to the column purification (pg. 4, 19).*

R1.C2
2. In line 193, the authors showed the number of CNAs in the single-time-point plasma samples from 8 CRPC patients. Besides the number of CNAs, the length of the genome affected by the CNAs might be more important, as CNAs have varied lengths. The authors should provide statistics about the length of CNAs.
*Response: We have included two new supplementary figures on the distributions of the CNA lengths in cfDNA across the 8 single time point prostate cancer patients (Fig. S3c-d) and added a discussion of these distributions to the main text (pg. 9, methods section pg. 22-23)*

R1.C3
3. To demonstrate the concordance of CNAs from plasma and metastatic tissue, the authors showed the correlated CNA burden (the number of CNAs). The number of CNAs alone could be too rough to prove the concordance, as the CNAs may have different breakpoints and different lengths. Besides the analysis of CNA burden, the author should compare the genomic position and the span of CNAs between matched plasma-tissue pairs.
*Response: We thank the reviewer for this valuable advice. We implemented another segmentation approach (multiPCF)[1] that detects common chromosome breakpoints across paired samples to find CNAs in both samples to calculate the correlation values. Using this approach, we normalized the CNA lengths by considering each CNA equally as events (regardless of genomic size) and computed Pearson correlations (table 1 below, column 2). While we observed small decreases in the correlation values when CNA segment sizes are normalized, we find these to be minor differences and the Pearson correlation values remain high for comparing plasma cfDNA to the metastatic tissues (irrespective of which method is used to calculate the correlations). The fairly consistent pearson correlation coefficient for the CBS and multiPCF approach suggests that the breakpoints and lengths of the CNAs are consistent between the plasma and metastatic samples. Therefore, we have retained the CBS based pearson correlation coefficient for further analysis.

Methods: Analysis of Genomic Copy Number Data from plasma DNA and Tissues using joint segmentation approach
The normalized read counts used for the CBS segmentation approach was used in the winsorize module of the copy number package to remove outliers in the copy number data using the default parameters. The data without outliers removed were segmented using multipcf[1] with the gamma penalty set to 10. Pearson correlation was used to understand the correlation between the segmented copy number plasma and metastatic samples.

sample_name multipcf_pearson_cor CBS_seg_pearson_cor
P5 0.763787099 0.744094
P6 0.637507484 0.820847
P7 0.567833061 0.645995
P8 0.931979319 0.947755
P9 0.912911422 0.917464
P10 0.77804446 0.840625
P11 0.90529671 0.853428
P12 0.912737457 0.911651
P13 0.739887638 0.61115
mean 0.794442739 0.810334333*


R1.C4
4. Line 506 - the authors said they used the correlation of the segmentation means to calculate the CNA correlation. The segmentation mean may refer to the mean depth of coverage within a segment. If true, then it is possible that the correlation was biased by the unmutated regions. The authors should justify this measurement.
*Response: We thank the reviewer for this advice and have calculated the Pearson correlation values using only the regions of the genome in which at least one samples has a CNA detected (by exclude diploid regions in both samples). While we observed a decrease in the correlation values for one patient (P5), we found that in most patients, only minor differences in the correlation values were observed when only the non-diploid CNA regions are compared (table 2 below). We have updated the Pearson correlation values based on these results in the revised text (pg. 9,10, methods section pg. 22, blue text).

sample_name all_segments_pearson_cor aneuploid_segments_pearson_cor
P5 0.744094 0.380044
P6 0.820847 0.803632
P7 0.645995 0.590055
P8 0.947755 0.939506
P9 0.917464 0.917236
P10 0.840625 0.841471
P11 0.853428 0.879121
P12 0.911651 0.907028
P13 0.61115 0.61492
mean 0.810334333 0.763668111*

Minor points:

R1.C5
1. Line 160 - the authors used "mutation frequency", which could refer to both mutation base frequency across the genome or mutation allele frequency. Probably the authors meant mutation allele frequency based on Fig. 1f. The authors should use a more precise word.
*Response: We have updated the main text and figures to replace ‘mutation frequency’ with ‘mutation allele frequency (MAF)’ as suggested by the reviewer.*

R1.C6
2. Line 179 - the authors used "cfDNA quantity", where they might mean "cfDNA concentration" based on Fig. S2e. The authors should use a consistent and precise word.
*Response: We have updated the word ‘cfDNA concentration’ and removed the term ‘quantity’ to be more consistent in the revised manuscript (pg. 6, 7, 8)*

R1.C7
3. Line 490 - the authors retained mutations labeled as "germline risk" by GATK's FilterMutect2Calls when they called somatic mutations. The authors should justify the reason to include these mutations.
*Response: The variants classified as ‘germline risk’ by mutect2 are borderline somatic/germline mutations. To better distinguish these calls, we applied additional filtering steps for read depth and alternate allele frequency, which removes the germline variants if they also occur in our matched normal tissue sample variant calls. Please see the description below for the ‘germline-risk’ variants from the MUTECT2 website, which we reclassify based on our matched normal filtering criteria. We have updated the methods section to clarify this point in our revised manuscript (methods section pg. 23).

https://gatk.broadinstitute.org/hc/en-us/articles/360037269791-Mutect2-BETA-*


Reviewer 2 Responses

Reviewer #2:
The manuscript by Ramesh et al. described a new approach enrich and profile plasma cfDNA for over 200 prostate cancer patients. They showed the amount of cfDNA is associated with aneuploidy and patient survival. For a small number of patients, genome and exome sequencing were performed to interrogate the CNV and mutation profiles. Furthermore, a subset of patients have longitudinal blood samples which were then used to infer clonal evolution upon treatment. As a non-invasive approach, cfDNA study is certainly of clinical significance and has received much attention in the past few years. This manuscript presented a thorough study of cfDNA in prostate cancer. In general, the data were clear and the manuscript was well-written. There were several places the authors failed to provide enough details or over-interpret the results. The authors have discussed the limitations of their approach, but some of their conclusions were misleading. The details are described below:

R2.C1
1. The first half of the manuscript focused on the associations between cfDNA level with many other factors. However, how sample processing step contribute to the variation of cfDNA level was not assessed. I wish the authors can extract the cfDNA from the same sample multiple times to quantify the variation between replicates. This would be a critical QC. In addition, when the authors used term cfDNA level, sometimes it referred to total cfDNA, sometimes concentration of cfDNA. What's the difference between these two measurements? If the input amount of blood is the same, these should be the same.
*Response:
To address this concern, we performed additional experiments in which cfDNA was isolated from 4 aliquots from the same plasma sample and compared the cfDNA concentrations after isolation using the same volumes. Our data suggest that there is only minor variation in the SEM of cfDNA concentrations between replicate samples isolated from the same plasma DNA tubes. We have added a figure to show these SEMs in the revised manuscript (Fig. S1e).

To clarify the differences between total cfDNA and cfDNA concentration,

cfDNA concentration (ng/ml)=(total cfDNA (ng))/(volume of plasma (ml))

Thus, the cfDNA concentration and the total cfDNA will be equal when the volume of plasma is 1 ml.*

R2.C2
2. In Figure1 and 2 as well as suppl. figures, the authors presented many associations between various factors and patient survival. Some were significant others were not. It is very hard to fully grasp all the information. It would be better to integrate all the factors into a Cox regression model and resolve the dependency. For example, both cfDNA level and aneuploidy were associated with survival. cfDNA level and aneuploidy were also associated. I wonder in a regression model, would both cfDNA level and aneuploidy carry independent predictive power.
*Response: To understand the intervariable dependencies we used 70 patients who had data available across all the variables considered. We performed cox regression analysis using cfDNA level, ploidy and all the genomic/clinical factors evaluated in the figures for overall and progression-free survival. Our univariate and multivariate cox regression models indicate that the ploidy of cfDNA has significant independent and joint predictive power for the overall survival. For the progression-free survival cox model, our multivariate analysis indicates that ploidy has significant joint predictive power. We have added a supplementary table with this information to the revised manuscript and a discussion in the main text (pg. 9, methods section pg. 25, Supplementary Table 3a-b).*

R2.C3
3. In method, the authors mentioned "Patients were classified as aneuploid or diploid based on the whole genome copy number data from the plasma". It seemed to be a very subjective judgement, not reproducible by other. What are the ploidy of samples shown in Fig 3 and 4?
*Response: To avoid human bias, we classified each CNA profile as aneuploid if it contains a large number of segments (>45) and has a deviation from the median segmentation value (>0.03) that is not explained by technical noise. If the sample has an intermediate number of segments (between 25 and 45), it is considered aneuploid if it has a deviation from the median segmentation value (>0.03) as well as a large segment or high segmentation value. All other samples are classified as diploid (methods section, pg. 19). We applied this method in our revised manuscript to reclassify all of the samples, that were previously classified manually as aneuploid or diploid.*

R2.C4
4. It is interesting to see that aneuploidy is associated with smaller DNA size. But I can't think of any possible explanation of how this could be.
*Response: The difference in DNA sizes has previously been reported in two studies [2, 3]. The smaller ctDNA size in tumors is thought to be due to more active transcription in cancer cells leading to more open nucleosomes. We have added these references to our revised results section (pg. 7)*

R2.C5
5. Based on the CNV and mutation patterns of P15 and P16, the authors used the term "genomic response to treatment". I don't think the observed pattern can be claimed as genomic response. A more likely case would be tumor stopped shedding DNA during that time. Or there might be something wrong with the sample.
*Response:
The fact that the changes in PSA levels correlate highly with the ‘genomic response’ or increase and decrease in exome mutation frequencies and the transition from diploid to aneuploid profiles argues that they do reflect changes in tumor volumes. PSA is a widely used blood biomarker that has been shown to correlate well with disease burden in prostate cancer patients. In Figure 5 and supplementary Figure S5, we can see that in most patients the PSA levels correlated very well with the changes in exome mutation frequencies and copy number profiles in both CSPC and CRPC patients (pg. 12,13).*

R2.C6
6. Page 12, "In these patients, the increase in genomic aberrations over time was likely due to increasing tumor purity". Why not increased tumor volume, or increased DNA release from tumor?
*Response: We agree with the reviewer that the increase in genomic signal from cfDNA can be attributed to any or a combination of factors such as tumor purity, tumor volume or increased DNA release from the tumor. We have added a discussion of these alternative possibilities to the revised paper (pg. 12, blue).*

R2.C7
7. The authors claimed "persisted through all of the timepoints". However, in P14, aneuploidy seemed to increase through treatment.
*Response: We agree that the purity of the tumor cells relative to the WBCs increases during the time points in this patient, however we observe the same CNAs and chromosome breakpoints (although more diluted), including a salient amplification of 8q(MYC) that is present before treatment (in addition to other events) in Fig. 5e.*

R2.C8
Some minor concerns:
1. Abstract format is not GB style.
*Response: we did not notice any issues with the formatting with our abstract for the GB style, which is the structured abstract.*

2. Words in title don't need to be listed as key words again.
*Response: we have updated these issues in the revised manuscript.

REFERENCES
1. Nilsen G, Liestol K, Van Loo P, Moen Vollan HK, Eide MB, Rueda OM, Chin SF, Russell R, Baumbusch LO, Caldas C, et al: Copynumber: Efficient algorithms for single- and multi-track copy number segmentation. BMC Genomics 2012, 13:591.
2. Mouliere F, Chandrananda D, Piskorz AM, Moore EK, Morris J, Ahlborn LB, Mair R, Goranova T, Marass F, Heider K, et al: Enhanced detection of circulating tumor DNA by fragment size analysis. Sci Transl Med 2018, 10.
3. Mouliere F, Rosenfeld N: Circulating tumor-derived DNA is shorter than somatic DNA in plasma. Proc Natl Acad Sci U S A 2015, 112:3178-3179.*

**Second round of review**

**Reviewer 1**

The authors' responses are satisfactory to me. However, before the manuscript would be accepted, the authors shall deposit all raw data and related clinical information, in order for the methods/results to be reproduced by others.

**Reviewer 2**

All concerns have been properly addressed.
